# Cross-disciplinary perspectives on the potential for artificial intelligence across chemistry

**DOI:** 10.1039/d5cs00146c

**Published:** 2025-04-25

**Authors:** Austin M. Mroz, Annabel R. Basford, Friedrich Hastedt, Isuru Shavindra Jayasekera, Irea Mosquera-Lois, Ruby Sedgwick, Pedro J. Ballester, Joshua D. Bocarsly, Ehecatl Antonio del Río Chanona, Matthew L. Evans, Jarvist M. Frost, Alex M. Ganose, Rebecca L. Greenaway, King Kuok (Mimi) Hii, Yingzhen Li, Ruth Misener, Aron Walsh, Dandan Zhang, Kim E. Jelfs

**Affiliations:** a Department of Chemistry, Imperial College London London W12 0BZ UK k.jelfs@imperial.ac.uk +44 20759 43438; b I-X Centre for AI in Science, Imperial College London London W12 0BZ UK; c Department of Chemical Engineering, Imperial College London London SW7 2AZ UK; d Department of Mathematics, Imperial College London London SW7 2AZ UK; e Department of Materials, Imperial College London London SW7 2AZ UK; f Department of Computing, Imperial College London London SW7 2AZ UK; g Department of Bioengineering, Imperial College London London SW7 2AZ UK; h Department of Chemistry and Texas Center for Superconductivity, University of Houston Houston USA; i UCLouvain, Institute of Condensed Matter and Nanosciences (IMCN) Chemin des Étoiles 8 Louvain-la-Neuve 1348 Belgium; j Matgenix SRL, A6K Advanced Engineering Center Charleroi Belgium; k Datalab Industries Ltd King's Lynn Norfolk UK

## Abstract

From accelerating simulations and exploring chemical space, to experimental planning and integrating automation within experimental labs, artificial intelligence (AI) is changing the landscape of chemistry. We are seeing a significant increase in the number of publications leveraging these powerful data-driven insights and models to accelerate all aspects of chemical research. For example, how we represent molecules and materials to computer algorithms for predictive and generative models, as well as the physical mechanisms by which we perform experiments in the lab for automation. Here, we present ten diverse perspectives on the impact of AI coming from those with a range of backgrounds from experimental chemistry, computational chemistry, computer science, engineering and across different areas of chemistry, including drug discovery, catalysis, chemical automation, chemical physics, materials chemistry. The ten perspectives presented here cover a range of themes, including AI for computation, facilitating discovery, supporting experiments, and enabling technologies for transformation. We highlight and discuss imminent challenges and ways in which we are redefining problems to accelerate the impact of chemical research *via* AI.

## Abstract 2

We offer ten diverse perspectives exploring the transformative potential of artificial intelligence (AI) in chemistry, highlighting many of the challenges we face, and offering potential strategies to address them.

## Graphical abstract

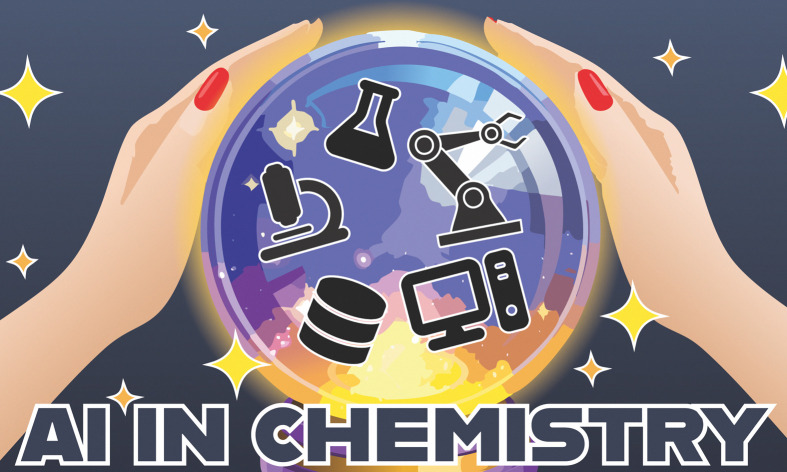

## Introduction

Artificial intelligence (AI) is undeniably revolutionising scientific research, enabling researchers to explore chemical phenomena at scales and speeds that would otherwise be unattainable. Indeed, chemistry faces several challenges that are well-suited to data-driven approaches; these challenges largely stem from the massive search spaces that chemists have at their disposal. Consider, for example, the vastness of chemical space; there are estimated to be 10^60^ candidate small organic molecules that could feasibly be synthesised. This does not account for the variety of methods, protocols, and procedures that may be used to make them, nor does it account for the number of subsequent materials for which they could serve as building blocks. Indeed, this ‘needle-in-a-haystack’ problem possesses many challenging layers, ranging from high-dimensional search spaces to many non-linear, often stochastic, relationships between structure and function. Yet, the ability of AI to assist in chemistry is not limited to searching chemical space; there is opportunity for AI to accelerate discovery through improving computational models, data characterisation pipelines, as well as providing support for automation of experimental methods.

The ability of AI to transform and accelerate research has been successfully demonstrated in numerous publications across chemistry. Indeed, these efforts have been highlighted in numerous reviews across sub-disciplines in chemistry.^1–7^ This recent surge in publications featuring AI to accelerate chemistry, in addition to the well-established chemical benchmarking datasets in the AI community, has led to collaborations across disciplines in ways not previously present in the literature. Ultimately, this has led to the application of state-of-the-art AI models to cutting-edge scientific research, featuring profound impact and implications for our ability to tackle complex scientific challenges.

With this collaboration between chemistry and AI, there is a beneficial increase in the diversity of perspectives within chemistry. Indeed, this discourse is prevalent across chemistry – from theoretical and computational chemistry, to experimental chemistry, as well as broader chemical initiatives that span research tools (*e.g.* molecular and materials discovery). Here, we present ten different perspectives on the impact of AI in chemical research coming from those with a range of backgrounds from experimental chemistry, computational chemistry, computer science, engineering and across different areas of chemistry, including drug discovery, catalysis, chemical automation, chemical physics, materials chemistry. The perspectives broadly covers the impact of AI on computation, discovery, experimentation, and its transformative role linking these through new technologies. We delve into the transformative potential of AI, highlighting many of the challenges we face, and offering potential strategies to address them.

## 1 AI for quantum chemistry

All of chemistry is an emergent property from the solution of the Schrödinger equation. As Dirac said, “the difficulty lies only in the fact that application of these laws leads to equations that are too complex to be solved.”

For nearly 100 years, quantum chemistry has seen the development of ever more accurate approximate solutions to the Schrödinger equation. These methods have grown in lockstep with the consistently exponential increase in digital computer power during the last eight decades. Due to electron correlation (which physicists call quantum entanglement), the direct exact solution of the Schrödinger equation scales with the number of electrons as 
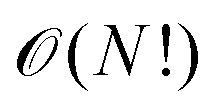
 (in this asymptotic ‘big O’ notation, the computational effort scales as the expression in the brackets). Practical quantum chemistry methods have, therefore, mainly been concerned with developing approximate solutions at a lower computational cost. These are often expressed on a Jacob's ladder from a fully correlated top rung (the ‘heaven of chemical accuracy’), to a totally uncorrelated bottom. Here, we discuss the impact of AI in quantum Monte-Carlo, density functional theory (DFT), and semi-empirical quantum chemistry.

### 1.1 Neural network wavefunction ansatz and quantum Monte-Carlo (QMC)

In principle, quantum Monte-Carlo calculations can evaluate a quantum-mechanical observable (such as the total energy) exactly with the usual stochastic error reducing as the number of samples 
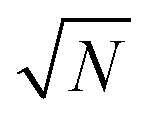
. In Bosonic systems, the errors are, therefore, under full control. In Fermionic systems (such as electrons in quantum chemistry), the fluctuating sign of the contributions to these integrals (due to the antisymmetry under exchange requirement of the Fermionic wavefunction, *ψ*(…, *r*_1_, …, *r*_2_, …) = −*ψ*(…, *r*_2_, …, *r*_1_, …)) exponentially slows down this 
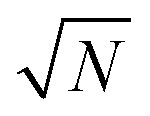
 convergence, limiting study to small systems and effectively imposing 
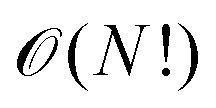
 scaling.

Adding knowledge about the wavefunction being integrated allows the use of importance sampling in the Monte-Carlo procedure. With perfect knowledge of the wavefunction, quantum Monte-Carlo becomes polynomial in time. Of course, if one had exact knowledge of the wavefunction, the Monte-Carlo calculation would be unnecessary!

One attractive aspect of quantum Monte-Carlo (and most post Hartree–Fock methods in quantum chemistry), is that they can generally be constructed in a variational manner. In a variational theory, any optimisation or adjustment which reduces the total energy takes the solution closer to the true value. This gives enormous freedom in the algorithmic approach to improve the solution, and a direct evaluation of the impact of any improvement. No external data is needed to evaluate the improvement, and so the approach can iterate between improving the guess to the wavefunction, and then using this wavefunction in Monte-Carlo evaluation of the energy. Such ‘self-play’ in the setting of symmetric games (such as chess) have enabled some of the most notable examples of superhuman AI.

The first application of neural networks (NNs) to represent many-body wavefunctions was by Carleo and Troyer^8^ in the context of a spin–lattice. To be used for quantum chemistry, this general approach needs to be extended to consider electrons in three-dimensional space.

There are two broad categories of quantum Monte-Carlo approaches:

1. Those constructed in first-quantisation directly consider the wavefunction over three-dimensional space *ψ*(*x*, *y*, *z*).

2. Those constructed in second-quantisation instead consider the wavefunction in terms of an occupation number over a finite basis, most often the Slater-determinants which result from a mean-field Hartree–Fock quantum-chemical calculation.

A notable benefit of first-quantisation is that no choice of basis set has to be made. Instead, the fundamental and general *ψ*(*x*, *y*, *z*) is being constructed. In systems where the chemical behaviour is not known *a priori*, this has the considerable advantage of not biasing the solution to what is expected. The complexity is that the methods used to predict the wavefunction have to correctly describe the antisymmetry present. A mean-field wavefunction in first-quantisation depends solely on the particle positions. Making the wavefunction dependent dynamically on the position of the other particles (configuration dependence) includes many-body correlation in the wavefunction. These are known as backflow wavefunctions, as the first application was the inclusion of an analytic hydrodynamic backflow contribution to the wavefunction in the study of liquid helium,^9^ which was later generalised and used to improve the solutions for electron gas calculations.^10^ The approach of using NNs to directly specify the backflow transformation^11^ with more flexibility then enabled with a fitted analytic model. PauliNet^12^ continues this approach further, producing a highly physically motivated NN with explicit Slater–Jastrow and Backflow components. FermiNet^13^ and Psiformer^14^ take a more maximally machine learning (ML) approach, giving more generalisability to the NN method. Both these approaches now enable state-of-the-art quantum Monte-Carlo calculations of small molecules (molecules featuring less than 100 electrons).

A major benefit of the second-quantisation is that the anti-symmetrisation of the wavefunction has been pushed into the use of Slater determinants (or mathematically similar functions); this means that the ML challenge can now use standard NNs and approaches. Here, one is trying to predict well-behaved occupation numbers of the second quantisation basis. A recent review focuses on NN ansatz in accelerating quantum Monte-Carlo calculations, with more technical detail.^15^

One issue with these techniques is that they tend to concentrate on the ground state energy of the system in question. From an experimental or molecular/material design point of view, this observable is not particularly interesting. Methods are much less developed, compared to quantum-chemistry approaches with a finite basis, to calculate response functions of the systems. There has been some interesting recent work in modelling excited states^16^ and unusual positronic chemistries^17^ with NN wavefunction approaches.

### 1.2 Machine learnt density functional theory (DFT)

The most successful method for quantum chemistry is density functional theory (DFT). These methods are based on the Hohenberg–Kohn mathematical proof that the same information that is present in the multi-dimensional electron wave function is equally present in the three-dimensional electron density. Practical Kohn–Sham implementations of this theory (where the kinetic energy is evaluated with a set of orbitals) rely on simple parameterisations for the correlation energy of the homogeneous electron gas, which in turn come from QMC calculations undertaken in the 1980s. The promise of ML approaches applied to DFT are that more powerful parameterisations could be developed which lead to more accurate solutions.

There are two mechanisms by which the machine-learnt DFT can be trained. The first is to use a training dataset, typically derived from a higher-level quantum-chemistry approach (such as CCSD). The functional is then modified to reproduce the reference energies (and sometimes densities). The second approach is to use the number of exact constraints on the electron wave function which can be analytically specified and, therefore, introduced into the training set used to train these more expressive functionals. These can be challenging to include into an interpretable analytic functional, but one can hope to correctly reproduce them with a more expressive machine learnt functional. An example is the fractional electron condition as used in the training of the DM21 functional,^18^ improving considerably on the fictitious charge delocalisation usually present in density functionals. Ultimately, developing ML density functionals is highly attractive, as there is considerable community expertise in using the techniques, and community codes in which the methods can be implemented.

Alternatively, orbital-free DFT dispenses with the Kohn–Sham orbitals to calculate the electron kinetic energy, and instead directly constructs kinetic energy as a functional of the density. This approach is more computationally efficient, as you avoid the 
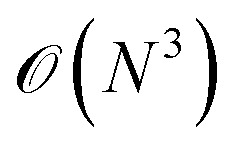
 cost of orthogonalising orbitals, permitting enormously large calculations compared to standard Kohn–Sham DFT. There has been some recent success in constructing this functional with NN approaches.^19^

### 1.3 Machine learnt tight binding (TB) and semi-empirical quantum chemistry

Tight-binding (TB) and semi-empirical quantum chemistry are the most simple (and therefore computationally efficient) models that directly represent the electronic structure of molecules and materials. These methods use a minimalist basis set (often just atomic orbitals), and include electron correlation effects *via* effective parameters. Thus, the methods typically scale with the 
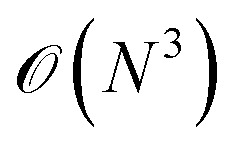
 cost of orthogonalising these orbitals. However, the size of the basis is much smaller than an *ab initio* basis set, and there are further methods, such as bond order potentials, which can use the same parameters without an explicit orbital representation. In order to simulate structure and dynamics, tight-binding models also require a (mostly repulsive) pair–wise interaction potential to prevent the atoms unphysically overlapping.

Two recent general tight-binding parameterisations that are seeing widespread adoption are the open-source DFTB (density functional tight-binding),^20^ and xTB (extended tight-binding)^21^ methods. These methods are semi-empirical, including atomic overlaps evaluated at the density functional theory level, with empirical parameters. Early work including machine learning in this area directly represented key steps in the Hamiltonian construction as a NN, enabling back-propagation of gradients, and, therefore, tuning.^22^ However, more recent work^23,24^ has generally used modern ML approaches (particularly, gradient optimisation and back propagation) to optimise standard parameters (based on the Slater–Koster analytic evaluation of atomic orbital overlaps), which enables a direct interpretation of the results and can more easily integrate with standard theoretical chemistry work processes. From this point of view, we can understand these approaches as building on the rich 80-year history of theoretical chemists building bespoke minimal parameter models, with the software-engineering and computational statistics of the big-data era.^25^

An alternative, hybrid, approach, is to use a delta-Machine-Learning technique to correct the results of a tight-binding model, which can be extended as a principal into a hierarchy of such corrections,^26^ or to use a tight-binding model as a computationally efficient way to provide a quantum-mechanically informed feature vector for a machine-learning model.^27^ However, research into these rather complex architectures has reduced recently due to the increasing power of force-field models.

A future development that is likely to have increasing importance is the use of equivariant and other more powerful basis sets (as developed for empirical ML potentials, see next section) to calculate the Hamiltonian. Zhang *et al.* applied the Atomic Cluster Expansion (ACE) to fit Hamiltonian matrix elements with greater accuracy and using less data.^28^ Generalising these methods to use a machine-learnt atomic feature vector would enable the construction of ‘foundational’ machine-learning tight-binding models of the entire periodic table.

### 1.4 Future outlook

There are unifying themes within the development of ML approaches for quantum chemistry. A motivating factor is that we can consider a quantum description of matter to be a strong inductive bias on the ML technique. Inductive biases lead to models that generalise better; by having a fundamentally quantum mechanical description, one would expect to have a model that has the correct long-range behaviour, and which extrapolates to larger systems.

Perhaps the most surprising aspect of applying ML to quantum chemistry, is that these methods have not come to dominate. So far, though the methods do offer improvements on the state-of-the-art, the gains are relatively marginal, and come with significant costs in terms of additional expertise required to undertake the calculations, and the underlying uncontrolled approximations inherent to the methods. Fundamentally, we have not yet seen a method with such a large performance increase (in terms of accuracy *versus* computation) that it dominates. In many ways, this mirrors the human-led development of DFT functionals, where the majority of researchers use relatively simple few-parameter functionals developed 30–40 years ago. One possibility is that this is an example of Sutton's Bitter Lesson,^29^ which states that more simple ML methods that can leverage larger amounts of data and compute will inevitably dominate in the long-term.

## 2 Scaling atomistic simulations with ML force fields

Simulating chemical systems at the atomic scale requires a model of how atoms interact. The traditional trade-off between accuracy and computational cost is being disrupted by ML techniques that combine the quality of quantum mechanical methods discussed in the preceding section with the speed of traditional interatomic force fields. There are several extensive reviews on this topic,^30–33^ but here we focus on some important developments and ongoing directions in ML force fields (MLFFs) for materials.

The potential energy surface of atomic configurations can be represented by empirical force fields – analytic models that approximate the forces between atoms as an expansion of two-body (distances) and higher-order (angles, dihedrals, *etc.*) terms. These models can be parameterised for specific systems (*e.g.* the TIP4P model for water^34,35^) or more general chemistries (*e.g.* the AMBER force fields for biomolecules^36^). Due to their fixed functional forms, such models are less accurate and transferable compared to quantum mechanical approaches. However, they allow simulations to be performed with length (nm–μm) and time scales (ps–μs) far beyond those accessible with methods such as DFT. The length and time scales afforded by these methods can describe rare processes (*e.g.* reactions in a catalytic cycle or crystal nucleation/degradation) and collective phenomena (*e.g.* self-assembly or spinodal decomposition), allowing for the study of emergent behaviours and material transformations.

### 2.1 Data-driven interatomic interactions

The training of force fields can be treated as a supervised learning task, where the input is the chemical structure. For crystalline materials, the outputs (labels) are usually the potential energy (*E*), atomic force (*F*), and cell stress (*S*). The quality and diversity of the *EFS* (potential energy, atomic force, and cell stress) training data determine the reliability of MLFFs in describing the thermodynamic and kinetic properties of compounds. For example, highly correlated systems (*e.g.* quantum spin liquids) would require labels from beyond-DFT methods, while polymorphic systems (*e.g.* perovskite crystals) require sampling of multiple structural configurations.

The architecture of an MLFF is defined by the combination of representation and regression. A structural representation that is equivariant with respect to geometric operations like rotations or translations is favoured to produce robust models that require less training data.^37–39^ Most representations start from atom-centred functions that describe the distribution of neighbours around a given site. Radial and angular basis functions are used in several schemes such as the smooth overlap of atomic positions (SOAP),^40^ moment tensor potentials (MTP),^41^ and atomic cluster expansion (ACE).^42,43^ The role of the regression model is to map between the structural representation and the *EFS* outputs. While early models were built on feed-forward NNs^44^ or Gaussian process regression,^45^ graph neural networks (GNNs) are now widely used including the open-source Nequip,^38^ Allegro,^39^ and MACE^46,47^ architectures.

### 2.2 Facilitating chemical insights

**Fig. 1:**
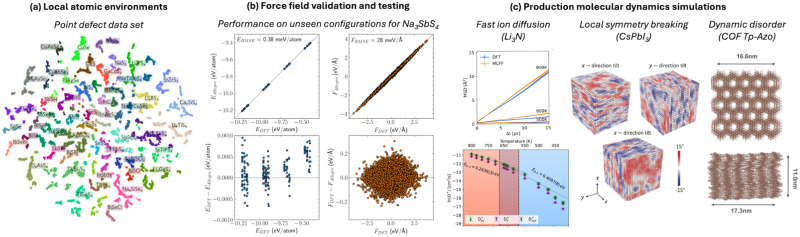
ML force fields involve (a) sampling of atomic environments (point defect dataset,^52^) (b) validation and testing on unseen configurations (parity plots and error distributions from an Allegro model,^54^) and (c) application to chemically interesting problems such as ion diffusion,^55^ symmetry breaking,^56^ and dynamic disorder.^57^ All figures are reproduced under a Creative Commons license.

The speed of MLFFs (typically 10^2^–10^3^ times faster than DFT) makes them attractive for use as surrogate models to tackle large compositional or configurational spaces, like crystal structure prediction^48^ or transition state searches.^49^ Beyond ideal systems, MLFFs have also been used to accelerate more realistic models of catalytic surface adsorbates^50,51^ or imperfect crystals. For example, a recent study of point defects trained a model on structural environments for 50 chalcogenide crystals (Fig. 1a) and showed a 70% reduction in the number of first-principles calculations required to identify the lowest-energy defect structure.^52^ More generally, surrogate models are being used to assess the structures, energies, and properties of novel compounds as part of materials discovery campaigns, which is the focus of the Matbench Discovery^53^ suite of benchmarks.

The scaling of these methods (often 
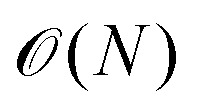
 with system size rather than 
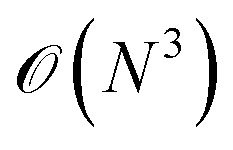
 scaling for standard DFT) enables compositionally complex systems to be tackled. For instance, the ionic conductivity of Na_3_SbS_4_ is enhanced when doped by W to create Na vacancies. A realistic description of systems such as Na_3−*x*_W_*x*_Sb_1−*x*_S_4_ would be prohibitively expensive using standard approaches; however, the Allegro architecture^39^ was used to train a predictive model (Fig. 1b) that was applied to system sizes up to 27 600 atoms for nanoscale-length simulations.^54^ Here, the test errors of <1 meV atom^−1^ approach the precision of the underlying DFT training data. Other examples from our work have included superionic phase transitions in Li_3_N,^55^ the formation of low symmetry phases in the halide perovskite CsPbI_3_^56^ and dynamic layer displacements in covalent organic frameworks^57^ (Fig. 1c).

### 2.3 Next-generation force fields

The development of MLFFs can be divided into several classes:

• Short-range potential. The first generation of MLFFs predict *EFS* by training on datasets derived from DFT calculations. The total energy of a system is expressed as a sum of the contributions from individual atoms 
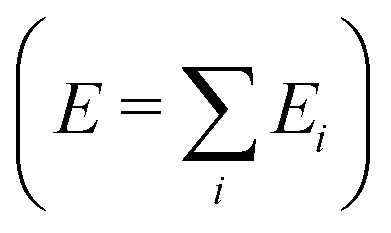
. The simple form has also allowed pre-trained foundation models for the entire periodic table^47,58–63^ with the number of parameters and performance listed on Matbench Discovery. Medium-range interactions (≈10–15 Å) can be captured through message-passing operations, which enable information exchange between neighbouring atoms.

• Long-range electrostatics. Explicit long-range interactions are essential in some cases; for example, in describing the electric double layer at solid–liquid interfaces. Electrostatic MLFFs have been developed that combine a short-range potential with an electrostatic potential (*e.g.* calculated using an Ewald summation). Ongoing developments are assessing different long-range descriptors^64^ as well as how charges are assigned (*e.g.* Mulliken, Hirshfeld) and redistributed during reactions, ranging from fixed point charges to charge equilibration schemes.^65–67^ The torch-PME package^68^ has been designed to support such developments by providing a framework to compute long-range interactions built on the PyTorch library.^69^

• Property prediction. There are ongoing efforts to extend predictions beyond energy and include other important physical properties, such as dipole moments,^70^ polarizabilities,^71^ electron density,^72,73^ wavefunctions,^74,75^ and even spectroscopic features.^76,77^ One notable frontier is the direct prediction of electronic Hamiltonians, which enables electronic studies of large-scale systems with the accuracy of hybrid DFT that would otherwise be prohibitive.^22,78,79^

MLFFs are quickly becoming essential tools in computational chemistry and materials science, enabling large-scale simulations over long timescales. Developments in more powerful model architectures, more diverse datasets,^80^ and the integration of uncertainty in both model training and deployment,^81^ are ongoing.

## 3 Generative AI

A major challenge in computational chemistry is the identification of molecules and materials with specific properties that are stable and synthetically viable.^82^ The most common discovery paradigm screens large libraries of known compounds for novel functionalities.^83^ ‘Inverse design instead starts with a target property and then aims to determine the specific atomic arrangement and composition needed to achieve it ref. 84. Genetic algorithms, particle swarm optimisation, random structure searching, and fragment-based screening can assist in the exploration of chemical space and to propose candidate structures.^85^

Generative AI offers a route to chemical discovery through probabilistic models that produce novel data.^86^ Driven by successes in inverse tasks across natural language, image, video, and audio generation,^87^ these methods are gaining increasing prominence in the chemical sciences. Crucially, they enable a natural coupling of structure generation with property constraints, directly allowing for the inverse design of molecules and materials.^88^ Despite the relatively early stage of development, a wide range of models have been trialled with varying success. While further innovations will be essential for practical use, these methods hold great promise for driving autonomous scientific discovery.

### 3.1 The evolution of generative models for chemistry

The key considerations of generative models for chemistry include (i) representation of chemical information in a latent space, for example, text or graph embeddings,^89^ (ii) inversion of the latent space to molecular and chemical structures, and (iii) generation of novel compounds and their properties through sampling a probability distribution.^90^

Initial approaches employed generative adversarial networks (GANs). GANs support property-guided exploration by modifying the generator with a multi-objective loss function^91^ or through combination with reinforcement learning to produce hybrid generative models.^92^ GANs have employed SMILES^93^ and graph representations^94^ for molecules, and compositional embeddings^95^ and 3D voxel grids^96^ for materials. In practice, training property-guided GANs is hampered by the sensitivity toward hyperparameters and the training protocol.^97,98^ Improved methodologies such as Wasserstein GANs can alleviate some of these issues and stabilise model training.^99^

Variational autoencoders (VAEs) aim to learn a low-dimensional representation of data (the latent space), through chemical encoders and decoders.^100^ Concurrently training a property prediction model to organise the latent space enables property-guided generation.^101^ The continuous representation also allows interpolation across chemical space. The first application of VAEs employed SMILES strings^102^ and graphs^103^ for molecules, while voxel grids^104^ and invertible crystallographic descriptions were applied for materials.^105,106^ VAEs enable property-driven molecular design through controlled modifications in the latent space (*e.g.* interpolations), making it possible to generate and optimize compounds with desired properties, a key advantage in molecular discovery.^101^ Outstanding issues include the high data requirements and the susceptibility toward discarding data variations.^107^

Similar to VAEs, normalising flows and diffusion models produce new data by sampling latent space. By applying an invertible (in normalising flows^108^) or stochastic function (in diffusion models^109^) they gradually transform noise into chemical representations. Initial implementations include GraphAF^110^ for autoregressive end-to-end molecule generation and CDVAE^111^ which combines VAEs with a diffusive decoder for crystals. Recent work has focussed on incorporating symmetry equivariances,^112,113^ property-guided generation,^114–118^ support for molecular fragments,^119^ and complex multidimensional constraints such as electrostatics and pharmacophores.^120^ Diffusion models have further been applied to accelerate structural relaxations by learning of smoother pseudo potential energy surfaces.^121^

Autoregressive Large Language Models (LLMs) (discussed further in later sections) generate data sequentially, with the transformer architecture being the most prominently used. Such LLMs enable the direct output of atomic structures in common text formats such as *XYZ*,^122^ crystallographic information files (CIFs),^123,124^ and SLICES.^125^ Fine-tuning of open-source foundation models can improve generation performance,^126^ and property-guidance is enabled through prompt-engineering.^127^ Many approaches have been investigated, from generating symmetry inequivalent units,^128^ to retrieval-augmented generation of known chemical libraries,^129^ and application of structured state space sequence models for drug design.^130^ Despite the inherent lack of invariances in text representations (*e.g.* permutation invariance) some models based on LLMs still achieve competitive performance.^131^ Hybrid models combining LLMs with other deep learning approaches are also common, including integration Riemannian flow matching,^132^ diffusion models,^131,133^ and contrastive learning.^134^

### 3.2 Challenges and opportunities

Generative modelling in the chemical sciences is still in its infancy, with many hurdles to overcome before it is used regularly for scientific discoveries. Below, we outline some of the main challenges and opportunities for the field:

• Beyond bulk materials and small molecules. To date, most attempts have been constrained to the generation of small molecules or crystals with less than 20 atoms in the unit cell.^135^ Technologically and pharmaceutically relevant compounds often are much larger and can contain disorder or defects.^136^ The field is already pushing in this direction, with generation of proteins,^137^ surfaces,^138^ porous materials,^139^ multi-component alloys,^140^ and metallic glasses.^141^ However, a recent benchmark showcased the limitations of existing models on interfaces and amorphous structures, highlighting the need for further developments.^142^

• Validation and benchmarking. Meaningfully evaluating the predictions from generative models is a major challenge.^135^ Metrics such as diversity and uniqueness are quick to evaluate but miss the main objectives of realistic and high-performance candidates. Similarly, structural validity assessment through charge neutrality and minimum interatomic distances is a poor proxy for kinetic and thermodynamic stability.^143^ Benchmarking platforms exist across the molecular^144,145^ and materials^146^ domains, however, an obvious gap is the absence of standardised multi-objective benchmarks for property-guided generation.^147^ While DFT has been employed for candidate evaluation^116,134^ it is computationally expensive and difficult to scale. Machine learned forcefield and property models offer an alternative route,^47^ but may be inaccurate for out-of-sample predictions.

• Synthesisability. The utility of generative models depends on their ability to suggest synthetically feasible candidates. One strategy involves human ranking of candidates to target in experiments.^148^ Another option is to bias generation towards accessible compounds by including a loss term for synthesisability.^149^ Challenges include the scarcity of widely applicable synthesisability metrics and the difficulty of balancing high-performance vs synthetic accessibility.^150^ A fully automated approach will be essential to enable closed-loop discovery. However, such platforms will likely have access to limited sets of reactants and processing conditions, thereby further constraining the range of accessible compounds with non-trivial impacts on synthesisability metrics.^151^

• Interpretability. Interpretable models are essential for expanding our understanding of the structure–property relationships across chemical space,^152^ and have implications for ethical and safe AI.^153^ State-of-the-art generative models are essentially black boxes and do not provide insights into why a particular compound was proposed.^154^ A key issue is the high dimensionality of embedded representations. While dimensionality reduction techniques can reveal the internal structure of latent spaces,^143^ they provide little information on the origin of proposed geometric arrangements. Emerging approaches for interpretable GNNs may be one strategy forward given their ubiquity in many generative models.^155,156^

### 3.3 Conclusions

Generative models have the potential to transform the Edisonian trial-and-error approach to chemical discovery. While the promise of efficient closed-loop workflows powered by generative models and self-driving experimentation is evident, generative approaches have a long way to come before this dream is realised. Few studies have reported the experimental verification of novel high-performing compounds proposed by a generative model. Success stories across biochemistry,^157^ antibiotics,^158^ and organic photovoltaics^159^ offer a tantalising glimpse of the impacts to come. However, as noted by Anstine and Isayev,^135^ even in failure, generative models can inspire human creativity and broaden our understanding of the chemical sciences.

## 4 AI for drug discovery

AI is already having a substantial impact on drug discovery,^160^ leading to improvements in overall pharmaceutical R&D productivity.^161,162^ Such productivity is expected to nearly double upon the successful completion of ongoing clinical trials.^160^ Here, we review the factors that have been contributing to this ongoing transformation with a focus on preclinical efforts, which account for over 43% of overall R&D expenditure.^163^ Indeed, while the preclinical stages of a successful project are less costly than the clinical stages, the high failure rate during preclinical development strongly contributes to overall R&D expenditure. For example, GSK reported data showing that 93% of their projects did not achieve an antibiotic drug lead, with half of the remaining 7% projects being stopped for not overcoming the remaining preclinical requirements.^164^ The latter is guided by ADMET (absorption, distribution, metabolism, excretion, toxicity) modelling of the drug lead, which is also important for reducing potential adverse effects in the subsequent clinical stages.

### 4.1 The impact of boosted funding

One key factor has been a substantial increase in private funding. Billions of pounds per year are invested in AI-driven drug discovery companies, sourced from partnership deals with pharmaceutical and biotechnology firms as well as private investors. This funding has enabled the development and prospective evaluation of AI models across drug discovery stages.

Another important factor has been sustained public funding, which supports the generation, collection, curation, and redistribution of data,^165,166^ along with the development of reusable software tools. This has resulted in a wealth of well-documented AI algorithms that can be combined with relevant domain knowledge.^167^ Notably, self-supervised learning algorithms, which pre-train deep learning models on large amounts of unlabelled chemical structures and then fine-tune them using much smaller labelled datasets of molecules.

Self-supervised learning is being used to build small-data AI models for drug lead discovery and potency optimisation. For instance, an LLM model pre-trained on over 77 million SMILES strings was fine-tuned to predict molecular properties.^168^ These approaches are also being adapted to leverage high-dimensional structured data.^169^ There are already proof-of-concept prospective studies for structure-based prediction of protein–ligand binding affinities using pre-trained language models.^170^ Similarly, GNNs have shown promise in phenotypic virtual screening, including applications to human pathogens^171^ and cancer cell lines.^172^ Beyond deep learning, methods for uncertainty quantification, such as Gaussian Processes^173^ and conformal prediction,^174^ have also proven their potential in this area.

Small-data AI modelling is also being investigated for a range of ADMET properties.^5^ For instance, using advanced feature extraction^175^ or multi-task learning to leverage data from similar molecules and/or properties.^176^ These studies typically build upon existing datasets and generic learning algorithms and further advance the field by releasing processed datasets and AI models for use in future projects.

### 4.2 The need for better benchmarks

A concerning trend is the proliferation, and often excessive hype, of publications describing new AI algorithms. While the number of these methods applied to retrospective benchmarks for drug discovery is rapidly increasing, only a small fraction of them have demonstrated their value in prospective applications. This highlights the need for benchmarks that are more closely aligned with the real-world demands of drug discovery. For example, MoleculeNet^177^ is a popular suite of benchmarks aiming at evaluating molecular property prediction (a relatively new umbrella term for virtual screening, binding affinity prediction or ADMET end point prediction). However, it is unrealistic for a number of reasons, *e.g.* using ROC AUC to evaluate performance in early recognition problems^178^ or employing unrealistically easy training-test data splits^179^ (scaffold split or even random split).

Progress in this area includes emphasising the use of more realistic data splits,^179,180^ employing performance metrics better suited to the specific tasks,^180,181^ leveraging true negatives in classification models to reduce false positives,^182^ and developing more comprehensive and representative benchmarks.^183^ These advancements will help address the gap between retrospective validation and prospective utility.

### 4.3 Recent technological breakthroughs expanding chemical and target spaces

Relatively recent technological breakthroughs have yet to reach their full potential. The first of them expands the chemical space *via* ultralarge libraries of molecules,^184^ synthesised on demand with success rates now exceeding 85%, which represents a major advancement.^185^ A key benefit of this technology is the unprecedented chemical diversity it provides, challenging the notion of an ‘undruggable’ target (was a target truly undruggable, or was the screened library simply too small to contain any drug lead?). The other key benefit is that screening larger libraries tends to yield a higher number of increasingly potent actives for a given target.^186,187^ However, a major roadblock is that screening the largest libraries remains accessible only to those with extensive computational resources, especially when docking is required to guide virtual screening. Encouragingly, new approaches are emerging to reduce the required resources,^188,189^ paving the way to democratise the screening of ultralarge libraries for any target.

The other technological breakthrough was AlphaFold2,^190^ which is expanding the 3D target space. AlphaFold represents a multidisciplinary effort combining AI, computational chemistry, structural bioinformatics, and well-aligned benchmarks. This method can predict the ligand-free 3D structure of a target from its amino-acid residue sequence. Therefore, it is particularly useful for the many targets lacking any experimentally determined 3D structure or even reliable homology models. Rigorous retrospective studies predicted AlphaFold2s utility for structure-based drug design,^191^ a prediction now validated in prospective applications as well.^192^ A far more challenging drug discovery application of AlphaFold3 to generate ligand-bound structures of unseen targets.^193^ The ambition is to be able to do this for any user-supplied molecule and target sequence without having to specify the binding site residues, with an output including the correct location, orientation and binding strength of the molecule. This has so far only been achieved partly for the handful of ligands well represented in the Protein Data Bank (PDB), which therefore form part of training sets complexed with seen targets. Many AI models building upon AlphaFold's principles have also been presented.^194^

### 4.4 The enhanced prospective performance of AI models

A growing number of prospective studies are revealing the immense potential of AI in drug discovery. For example, GNN models have been used to identify novel molecules with whole-cell activity against *E. coli*,^195^*A. baumannii*^196^ or *S. aureus*.^197^ The discovery of antibiotics for these drug-resistant pathogens is both urgent and critical in addressing the antimicrobial resistance crisis.^171^ AI models for drug response prediction are also advancing in other disease models, while ML scoring functions for structure-based drug design have made significant strides since their inception.^198^ Among these, virtual screening remains their most challenging and impactful application.^199–201^ AtomNet, an ensemble of convolutional NNs, is currently the most successful ML scoring function for virtual screening.^202^ Atomwise, the company behind AtomNet, applied it prospectively on 318 targets as part of their Artificial Intelligence Molecular Screen (AIMS) programme. This ambitious effort involved partnerships with 482 academic labs and screening centres from 257 institutions across 30 countries. Despite focusing on hard targets and testing only molecules dissimilar from known actives, submicromolar actives were identified in approximately 60% of targets through dose–response experiments. Remarkably, this was achieved by synthesising and testing an average of just 85 molecules per target.^202^

### 4.5 Next steps

To make AI-driven drug discovery more resource-efficient, future research must focus on improving our ability to create retrospective benchmarks that reliably predict prospective success. These benchmarks are critical, as they guide the selection of AI models for prospective applications. Another roadblock is data being repurposed from often heterogeneous datasets originally generated for different objectives, which introduces several issues, such as bias, inconsistency, or limited data size.^203^ A promising recent trend involves generating training data for the target using the same experimental protocol later employed to validate prospective predictions *in vitro*.^195–197^ This could circumvent some of these issues in AI for drug discovery.

## 5 Synthesis route planning and selection *via* deep learning

With recent advances in generative ML, it has become possible to computationally design promising molecules for diverse applications in the (bio-) chemical and medicinal sciences. Whenever a novel molecule, for example a candidate drug molecule, is designed or generated (*via* ML), it must pass the synthesisability test. In other words, it does not matter whether one can computationally design the perfect molecule in terms of properties, if it cannot be synthesised in the laboratory, the molecule will always remain virtual.

### 5.1 Retrosynthetic search

The first requirement to produce the real molecule through experimentation is a synthesis path connecting the molecule (product) to purchasable building blocks through a series of reactions. This path, also known as a synthesis route, is traditionally mapped out by experienced chemists in a backwards fashion – known as retrosynthesis.^204^ Retrosynthesis is a laborious and tedious process that highly depends on the chemists’ expertise for specific reaction types.

To automate retrosynthesis, Corey *et al.*^205^ encoded reaction rules into a machine-readable format. His pioneering work in 1972 led to the development of expert systems that perform retrosynthesis in an autonomous fashion.^206^ With increased applications of ML in chemistry, the rule-based systems have slowly been outnumbered in favour of deep-learning approaches.^207^ Below, we provide a short overview, outlining current challenges and opportunities.

**Fig. 2:**
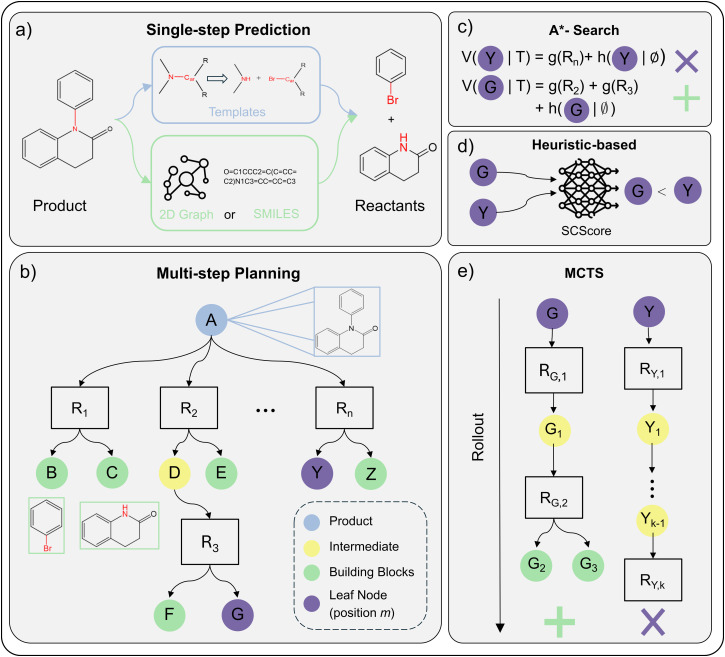
A holistic overview of ML-driven retrosynthesis (Section 5.1) (a) example of performing single-step reaction prediction on a product molecule. The reaction is either predicted *via* reaction templates (template-based) or in a data-driven fashion using SMILES or 2D Graphs as the molecular featurisation (semi-template/template-free). (b) AND-OR search tree for multi-step planning. *R*_*i*_ denotes a specific reaction that is applied to the parent node (molecule). Children nodes are precursors to *R*_*i*_. Leaf nodes (purple) are open positions *m* in the tree that will be expanded by the single-step model. (c)–(e) Strategies for leaf node (position m) selection/prioritisation. Subplots assume same retrosynthesis tree as shown in *b*. Furthermore, we assume that node *G* is preferable to node Y. (c) A*-Search calculates the value of the open position *m* as a sum between reaction cost *g*(*R*_*i*_) and future cost *h*(*m*|∅). As *G* is assumed to be preferable, *h*(*G*|∅) ≪ *h*(*Y*|∅) and/or *g*(*R*_2_) + *g*(*R*_3_) ≪ *g*(*R*_*n*_) (d) heuristic-based search uses a pre-defined heuristic to assign a value. In this example, we assume that the SCScore^213^ heuristic prefers position *G* over *Y*. (e) Monte-Carlo Tree Search traditionally uses rollout. For node *G*, the rollout leads to building blocks *g*(*G*_2_) and *g*(*G*_3_). For *Y*, the rollout is unsuccessful and terminates at after *k* sampled reactions. Thus, the reward is given to position *G* and it is preferred for selection.

Data-driven retrosynthesis is constituted of two distinct parts: (i) single-step predictions^208,209^ and (ii) multi-step route generation.^210,211^ Single-step models predict a single-step reaction, that is, to find plausible reactants for a given product. Multi-step algorithms apply single-step models recursively to build synthesis routes of *N* single-step reactions ending in a set of purchasable molecules. Combining these two parts, one can generate several synthesis routes for a single product, referred to as a synthesis tree.^212^Fig. 2 provides an overview of this concept, showing how a target molecule can be broken down into multiple possible precursor combinations.

#### 5.1.1 Single-step prediction

Over the years, three distinct branches have emerged for single-step prediction:

First, template-based models utilise reaction rules.^214–218^ These rules define which bonds to break in the product and leaving groups to attach within the reaction centre (Fig. 2a). Reactions rules are usually obtained from literature precedence.^219^ Recently, researchers explored generative approaches to create reaction template, overcoming the limited reaction space covered by literature precedence.^220–222^

Second, semi-template models mainly split the predictions into two sub-tasks of reaction centre identification and synthon completion.^223–227^ The model identifies the reaction centre as the atom and bonds participating in electron rearrangement in a reaction.^228^ The output of this first step is a group of molecules (synthons)^224^ that are not chemically valid. To validate the synthons, the second step adds atoms^224,229^ or motifs/leaving groups^230,231^ to the synthons iteratively. Upon completion, a set of reactants is returned. Since semi-template models perform direct edits on the molecular graph, GNNs are the preferred ML architectures.

Third, template-free models perform sequence translation to generate the reactants token-by-token, mainly in their SMILES strings.^232,233^ The input to the model is either the product SMILES^234–236^ or 2D molecular graph.^237–239^ Since the nature of the problem is generative, the (graph) Transformer is the preferred.^232,238^ Furthermore, owing to its generative nature, template-free models can also predict reagents (solvents/catalysts) in addition to reactants given only the product.^232,240,241^

However, the combined problem of reactant and reagent prediction is difficult in nature. Most single-step retrosynthesis algorithms therefore focus on predicting reactants. Following the prediction, a separate (standalone) model recommends suitable conditions such as yield,^242^ catalysts, solvents, and temperature, framed as a multitask prediction problem.^243,244^ Unlike the combined problem, the reaction condition model takes the full reaction string (reactants → products) as input.

#### 5.1.2 Multi-step search

To ensure that the synthesis routes are promising, the single-step models are guided by search algorithms.^210,211^ These algorithms ensure that all precursors for the synthesis plan are purchasable. Furthermore, the algorithms return the synthesis routes within a certain time and call budget, reducing the computational cost.

The search algorithms construct a synthesis tree/graph *T* with the product molecule as the root and purchasable molecules as terminal leaf nodes. Each branch of the tree is a distinct synthesis route consisting of several single-step reactions. The single-step reaction are represented in the tree by AND nodes. The parent of the reaction node is the product (outcome) of the single-step reaction. Precursors to the reaction are added as children (OR) nodes. This AND-OR assignment of nodes follows intuition: for a reaction to happen, all precursors must be available (boolean AND). On the other hand, a molecule can be synthesised as long as there exists one feasible reaction (boolean OR). For other types of retrosynthesis trees/graphs (*e.g.* OR tree), we refer the reader to previous publications.^210,240^

Fig. 2b shows an example of a partially explored synthesis tree. The algorithm constructs the tree/graph by selecting a (non-terminal) leaf node *m* and querying the single-step model to propose additional *n* reactions along with their precursors. The precursors are then added to the tree/graph, referred to as expansion. In Fig. 2b, node *G* or *Y* would be expanded next by the single-step model.

Most importantly, the search algorithms should be able to discern a good leaf node (position) *m* in *T* from a bad position. A good position is ideally expanded and exploited, while a bad one is abandoned. In other words, one wants to explore promising reaction pathways instead of wasting resources on potentially unfeasible pathways. For this purpose, researchers have proposed three different strategies: Monte-Carlo Tree Search (MCTS),^210,245–247^ A*-Search,^211,248,249^ and heuristic-based exploration.^240,250^ Below, we describe the node selection strategies for open positions *m* in *T*. From a synthetic chemistry perspective, this involves choosing which intermediate molecules *m* in the synthesis pathway should be prioritised for further retrosynthetic analysis. Some intermediates may resemble readily available commercial compounds, while others require additional synthetic steps to reach simpler precursors.

• MCTS evaluates position *m* by rollout (iteratively expanding a node until termination).^210^ Rollout iteratively samples random reactions from the single-step model (Fig. 2e). If the “random walk” terminates in purchasable building blocks, a reward is assigned to position *m* rendering *m* preferred to be expanded. As rollout is computationally expensive, and researchers proposed to use a model (usually a NN) trained from experience^247,251^ to replace rollout.

• A*-Search evaluates position *m* using a value function combining the cost of reactions in the existing tree (*g*(*m*|*T*)) with an estimated cost of future reactions (*h*(*m*|*T*)) as shown in Fig. 2c. As the cost of future reaction is not known for leaf nodes *a priori*, one must approximate it as *h*(*m*|∅) (the cost of synthesising *m*).^211^ Using cost estimates from existing synthesis trees, one can learn *h*(*m*|∅) in a supervised fashion.^211,248,249,251^ Otherwise, seeing the retrosynthesis planning problem as a single-player game,^252^ one can learn *h*(*m*|∅) online using self-play, also referred to as reinforcement learning.^253,254^

• Heuristics-based evaluates a position, as the name suggests, on user-defined search heuristics. Popular heuristics include accessibility metrics such as SCScore^213^ (Fig. 2d) or SAScore,^255^ the overall route length or molecule disconnection preferences.^246^

The search guidance provided by these algorithms definitely biases the search towards purchasable building blocks, but there is no theoretical guarantee that these will in fact be reached. Recently, Yu *et al.*^256^ proposed bi-directional search to alleviate this problem. By simultaneously building two synthesis trees, one going backwards from the product and one going forwards from the building blocks, they ensure constraint satisfiability.

The constraint of synthesis route feasibility is harder to achieve. Herein, feasibility refers to the likelihood of the synthesis plan to be validated through experimentation. This is because vital information such as yield, selectivity and reaction conditions are generally missing from the synthesis plan. Tripp *et al.*^257^ addressed this shortcoming by changing the search goal to (most probably) include at least one feasible synthesis route in the synthesis tree. Another idea is to propose synthetic routes that closely resemble existing routes in literature.^258^

#### 5.1.3 Open-source software

For ease-of-use, several open pieces of retrosynthesis software have been developed, integrating single-step models with multi-step search algorithms. Amongst them, AIZynthFinder,^245,259^ ASKCOS,^260^ and IBM RXN^261^ are popular. AIZynthFinder can be accessed *via* GitHub, while ASKCOS and IBM RXN are hosted on their own websites. ASKCOS and IBM RXN provide a user-friendly GUI that does not require any form of coding knowledge.

### 5.2 Challenges and opportunities for synthesis planning

Rapid advances in the field of retrosynthesis planning has triggered the development of an overwhelming choice of different model architectures. Despite this, the community needs to overcome several challenges to yield a fully functional retrosynthesis tool.

• Interpretability and reliability. Arguably the key cornerstone to a successful synthesis framework is the reliability of the single-step model. A reaction proposed by the model should ideally yield the product through experimentation. However, researchers often evaluate single-step models using model recall, known as top-*k* accuracy. By only focussing on recalling the reaction in the existing database using top-*k* accuracy, one can easily forget about the quality of all other reactions proposed by the model.^207,262^ This is indeed a problem, since the single-step model adds *k* reactions to the multi-step search tree during each expansion phase. To improve the reliability of the single-step model, one could use a post-hoc filter removing poor reactions.^210,240^ Otherwise, during model training, one can augment the dataset with negative (non-feasible) reactions.^222^ While both methodologies can improve the reliability, they are not rigorous. A more rigorous approach would build on thermodynamic insight, exemplified by the work of Ree *et al.*^263^ To further increase interpretability in the single-step models, one could augment the predicted global reaction with mechanistic insight.^264^ Lastly, researchers should prioritise evaluating their ML models on existing benchmarks^207,262,265^ and reconsider the overreliance on the top-*k* accuracy as a performance metric. Standardising evaluation practices not only facilitates the identification of model limitations, but also promotes transparency and clarity in reporting.

• Route selection strategies. Little research has addressed the (post) selection of synthesis route following the multi-step search. The multi-step algorithm returns *N* different synthesis routes to the end-user, where *N* depends on the time/iteration budget. The search algorithm ranks these routes by considering route length, number of reactions and/or overall route cost (single-step confidence).^245^ However, these are not clear indicators to confidentially claim that one route is better than another. Unfortunately, one does not have access to informative metrics such as overall route yield or the actual (physical) cost of carrying out the reactions. Badowski *et al.*^266^ assumed a fixed yield and fixed cost for each reaction, circumventing the problem. Fromer and Coley^267^ propose to select synthesis routes that maximise the utility of synthesising a batch of molecules (*e.g.* for virtual screening routines). Yujia *et al.*^268^ trained a model to select synthesis routes that are most likely feasible according to human expertise. All these approaches are good starting points and can be extended by considering other factors such as route ‘greenness’^269^ and scale-up potential.^270^

• Sustainability. Instead of selecting green routes *a posteriori* as suggested above, researchers have attempted to bake in sustainable aspects into the retrosynthesis framework. The first idea is to include biosynthetic/enzymatic reactions in the single-step model^271,272^ to bias search towards sustainable, energetically favourable reactions. Another idea is to preferably select routes utilising green solvents within reactions.^273^ As we strive towards greener chemistry, this field of research holds a lot of promise, and yet, there are still several challenges to address.^274^

• Implementation and adoption. Implementation and adoption is eased with existing open-source software and user-friendly interfaces.^259,260^ Nonetheless, the amount of papers reporting the synthesis of novel and/or complex molecules^264,275^ remains limited. This is partly attributed to a lack of interpretability and reliability, as discussed above. As models become more reliable in the future, we can expect increased adoption by scientists.

• Data sources. All points mentioned above are somewhat dependent on an improvement of current datasets and better data availability. Predominantly, the open-source USPTO database is used for model training and testing.^276,277^ The database is known to be scarce in terms of reaction conditions, often not reporting reagents, yields and selectivity.^278^ Commercial databases such as Reaxys^279^ are well-documented containing millions of substances and reactions, but are locked behind paywalls. This led to the development of collaborative initiatives to build a database through community engagement. Most well-known is the Open Reaction Database (ORD),^278,280^ which encourages chemists to contribute and upload their datasets. This initiative is still in its infancy and most of its entries are currently from the USPTO. Better awareness and integration is therefore needed to improve current databases.

## 6 Data-rich and data-led experimentation to support development of accurate and predictive models

**Fig. 3:**
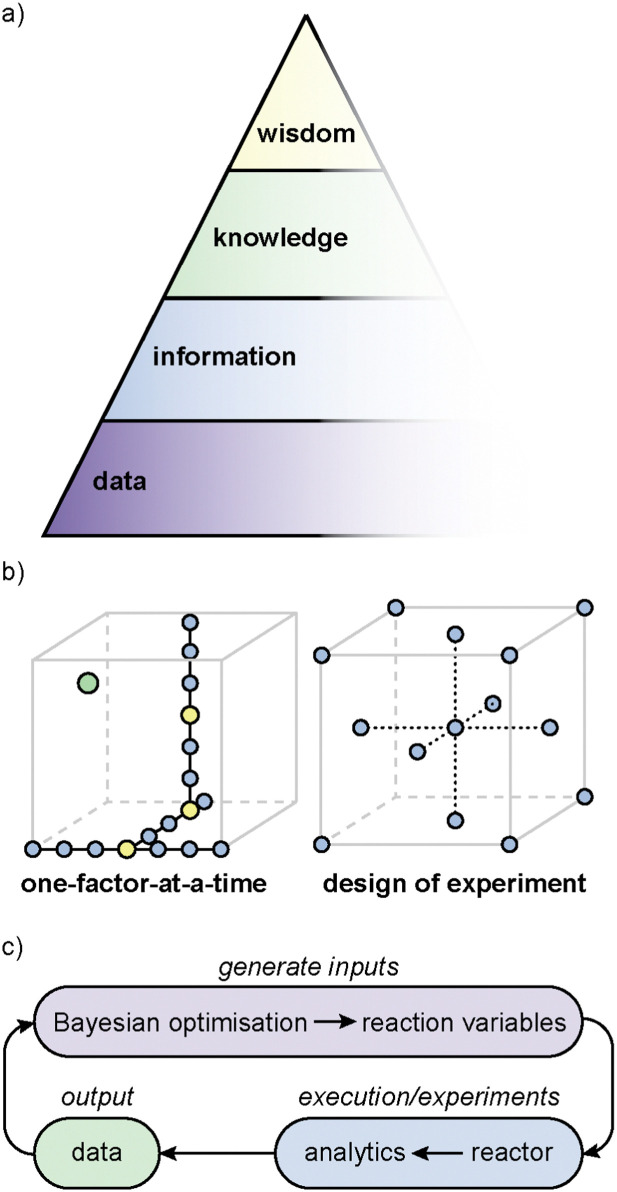
(a) Data-information-knowledge-wisdom pyramid. (b) One-factor-at-a-time (OFAT) and design of experiment (DoE) approaches to optimisation. The optimal point is shown in green, and the points that are the optimal along a certain parameter are highlighted in yellow. (c) An example closed-loop optimisation workflow.

The Data, Information, Knowledge, Wisdom (DIKW) hierarchy (Fig. 3a), also known as the knowledge pyramid, is widely evoked in AI as a model to represent the progression from data to wisdom.^281^ At the base of the pyramid is (raw) data, which may consist of unprocessed facts and figures. As we move up the pyramid, data is organised and classified; transforming into information that can subsequently be analysed to afford understanding and insights (‘knowledge’). Finally, at the pinnacle of the pyramid is wisdom, where the knowledge is applied to make informed decisions.

In ML, data is needed to train, validate and improve the AI model. The accuracy and reliability of AI depend on the availability of data collected from experiments. For the chemical sciences, there are generally two types of data:

1. Compound (characterisation) data. Typically collected after the reaction with the isolated compound or simulated computationally *in silico*. These data are needed to confirm the chemical composition and structure, as well as physical and chemical properties. There are many different types of characterisation data, ranging from discrete values (*e.g.* melting/boiling points, bandgap energy, emission wavelength), a dataset, or ‘fingerprint’ (*e.g.* NMR, IR spectroscopy), or images (*e.g.* morphology and particle sizes recorded using microscopy). Accordingly, these data are also highly heterogeneous, by nature. Characterisation data are closely associated with the molecular composition and structure, they are particularly useful for the prediction of chemical/physical properties, *e.g.* for the discovery of new materials; or biological properties, for applications in the discovery of new drugs, for example.

2. Reaction data. This is acquired during the reaction using either *ex situ* or *in situ* quantitative process analytical tools to monitor either the rate of formation or consumption of reaction components (typically reactants and products). The temporal progress of the reaction (kinetics) is particularly important to understand reactivity. Kinetic data is essential to elucidate reaction mechanism, enzyme kinetics (for designing more effective drugs), material degradation (improve safety and sustainability), and development of a commercially viable process (scaling up), for example. In contrast to characterisation data, kinetic data are much more uniform and ‘continuous’ in nature (either concentration *vs.* time or rate *vs.* temperature). A chemical reaction also involves several closely associated discrete and continuous variables that synergistically influence the reaction outcome; for example: reactants stoichiometry, catalyst, pH, additives, solvent, temperature, and pressure. The ‘robustness’ of a chemical reaction denotes its capacity to withstand variations in these variables without detrimental effects. Understanding the impact of these variables on the reaction outcome is essential not only for elucidating the reaction mechanism, but also for designing a process that ensures consistent product quality (‘Quality-by-Design’, QbD), which is particularly important for highly regulated industries such as pharmaceutical products.^282^

Traditional practices in chemistry have long relied on hands-on experimentation and observation, where experimental design and the interpretation of the results are still largely based on ‘chemical intuition’, acquired through empirical observations over many years (‘experience’). Under such conditions, workflows often follow one-factor-at-a-time (OFAT) experiments, where the effect of one factor (or variable) is studied while keeping the other factors at fixed values (Fig. 3b). Although this approach can be effective for optimising the yield of a simple reaction, it does not take into account interdependencies between the reaction variables, and could potentially miss the optimal point (Fig. 3b, green dot indicated). As the end point is arrived at empirically, it is impossible for such an approach to be able to predict the outcome of similar reactions; neither can it tackle multi-objective optimisations.

In the past decade or so, there has been increased interest in the use of statistical methods for optimising chemical processes. One of these is design of experiments (DoE), a popular statistical method that can be used to interrogate relationships between the reaction parameters (‘factors’) and outcomes (‘responses’), systematically (Fig. 3b).^283^ The approach starts with identifying the objective, which could include maximising yield, improving selectivity, or shortening reaction time, *etc.* The researcher then determines the factors that might influence these outcomes (‘responses’). Common factors include temperature, reactants concentration, catalyst loading, choice of solvent and reaction temperature. Using either a full- or partial-factorial design, different combinations of factors and levels are generated, and the experiments are randomised to minimise the effect of uncontrolled variables (for example, catalyst deactivation). The responses are analysed using Analysis of Variance (ANOVA) or regression analysis to produce *F*- and *p*-values against each factor, and also combination of factors (quadratic). If the *F*-value is significant and the *p*-value <0.05, the specific factor, or combination of factors, are considered to be statistically significant. The model is then used to produce a validation set of experiments, which is performed to test the accuracy, before the model is used to predict the final optimal outcome, which can be a balance between different objectives. In recent years, DoE has been applied successfully in optimising several chemical processes.^284,285^ Typically, DoE can require a large number of experiments, which can be costly and time-consuming. However, this has been largely addressed by advances in laboratory automation, to enable high-throughput experimentation^286^ and analytical techniques.^287^

In contrast, Bayesian optimisation utilises a surrogate model (typically a Gaussian process) to approximate the objective function and an acquisition function, to determine the next experiment to perform.^288^ This is particularly suitable for high-dimensional problems and also provides a measure of uncertainty. Bayesian optimisation will require a ‘close-loop’ experimentation, where the predictive algorithm is integrated with experiments on an autonomous robotic platform (or ‘self-driving lab’) (Fig. 3c).^6,289^ While this may minimise the number of experiments, it is also computationally more expensive. Furthermore, the integration of expert knowledge is also often needed to select the appropriate parameters to fine-tune the black-box functions.^290^

## 7 LLMs and multimodal models for chemistry

Large language models (LLMs)^291–293^ represent the latest ground-breaking developments in the field of natural language processing (NLP) with profound impact in general AI research. LLMs are pre-trained on web-scale corpus of text data (including natural language text and code, *etc.*) with the goal of learning rich internal representations of documented knowledge during human history.^294,295^ After pre-training, LLMs can then be used as a general-purpose AI for a diverse range of downstream tasks (such as sentiment analysis^296^ question-answering,^297^ and generative tasks,^298,299^) *via* fine-tuning on downstream task data, and/or in-context learning where the model learns to solve a task purely from the relevant context provided by the prompts.^300^ Furthermore, the ability to interact with an AI model *via* human language allows for descriptions of more complex tasks and reduces the barrier of AI expertise for using powerful AI models.^301,302^

### 7.1 Chemical representations with LLMs

LLMs have attracted increasing attention in AI for chemistry research through the development of so-called “molecular” or “chemical” language models.^168,303–305^ These models largely inherit the transformer-based network architectures^292,295^ and pre-training strategies from popular LLMs in the NLP domain, except that they operate on text-based chemical data, such as SMILES strings.^306,307^ Similar to natural language-based LLMs, molecular LLMs learn to represent the underlying structural properties of molecules by understanding the unique grammar of chemical textual data. This again enables diverse applications with pre-trained molecular LLMs, including molecular property prediction (MPP),^168,303,308^ conditional molecule or material structure generation,^304,309,310^ and retrosynthesis.^233,311,312^

### 7.2 Multimodal models for chemistry

Though increasingly used, current molecular LLMs fall short in fully representing the structural information and equivariance properties of molecular data. In general, molecules are challenging objects to describe: for instance molecular orbital theory^313^ and valence bond theory^314^ offer contrasting descriptions of a molecule. Therefore, limiting the representation of a molecule to a single form will not capture the full behaviour of the molecule. This motivates the creation for multimodal frontier models that can capture richer representations of molecules^315,316^ by incorporating molecular graphs, coordinate information, sequential data and other features to improve their performance on various downstream tasks such as property prediction.^305,317–320^

### 7.3 LLMs for scientific workflows

LLM-assisted workflows are of particular interest for LLM developers and users since ChatGPT's release.^321,322^ In particular, LLMs are efficient at digesting, summarising^323,324^ and retrieving information from large documents;^325,326^ question-answering from prompt inputs;^297^ as well as performing domain-specific tasks such as translation,^327^ and computer programming.^328^ Also, very recent developments regarding reasoning and planning complex tasks with LLMs have shown promising results.^329–331^

The diverse capabilities of LLMs offer many exciting opportunities in improving the workflows of scientific research in chemistry domain. To understand how LLMs can assist scientific discovery, an analysis of essential workflow steps with potential uses of LLMs is provided below.

• Idea formulation. LLMs trained on scientific publications and chemistry textbooks can assist scientists in formulating innovative research ideas. *Via* prompting^332^ and retrieval augmented generation techniques,^333,334^ LLMs can efficiently retrieve and summarise existing scientific knowledge in published/proprietary documents regarding a scientific question of interest.^335^ This qualitative information obtained *via* LLM-assisted search complements the quantitative information extracted from existing data-mining tools for chemistry data,^336,337^ contributing to a holistic overview of the scientific question to be addressed.

• Lab experiment troubleshooting. With access to electronic lab notebooks and chemistry literature, a domain-specific LLM can be used to troubleshoot specific issues during the lab experiment process.^338^ The natural language understanding capabilities of LLMs are especially useful in analysing text descriptions of experimental conditions that can vary in style across documents. Also, anomalous results may be described to an LLM *via* natural language, where the LLM can then retrieve relevant papers describing similar issues and provide a natural language explanation for the results. LLMs plays a growing role in teaching,^322^ and this may extend into the lab where students can use LLMs as a resource to aid in laboratory technique and troubleshoot issues.

• Experiment design. With access to external computational tools, LLM-hybrid models can perform complex planning tasks,^339^*e.g.*, AlphaProof and AlphaGeometry^340^ for solving mathematics problems. Thus an LLM can be prompted to suggest plausible experimental procedures based upon existing lab data, for which scientists can then verify within a lab. LLM-assisted planning can complement existing experimental design algorithms (*e.g.*, Bayesian optimisation^341^): the former can better utilise scientific knowledge and data presented in natural language form,^342^ while the latter can provide precise quantitative parameters for setting up the lab experiment. Fully autonomous chemical research may be possible with LLMs planning the high-level experimental steps (*i.e.* a sequence of action primitives)^343^ and autonomous lab robots executing the planned actions.^344,345^

### 7.4 Challenges for LLMs and frontier models in chemistry

Despite the aforementioned exciting opportunities, a number of profound challenges remain to be solved for training and utilising LLMs and frontier models for chemistry research and applications.

• Mitigating hallucinations. LLMs are prone to hallucinations – they can generate responses that do not make sense for the given task.^346,347^ In molecular generation, hallucinations can lead to invalid molecular structures: for example, an atom having too many bonds.^348^ Hallucinations can also lead to inconsistent results when retrieving scientific information from research documents *via* LLMs. Efficient mitigation of hallucinations is key for the reliability of LLM usage in chemistry, *e.g.*, experimental conditions should be retrieved precisely without removing important information or adding false data.^349^

• Data collection and data-efficient training. A critical challenge in modelling molecules is the high complexity of the data space containing all valid molecules. For instance, activity cliffs exist in such space, whereby a small change in molecular structure can result in large changes in molecular properties.^350^ Therefore, labelled data collection (with large quantity and high quality) remains a major bottleneck in molecular property prediction and generation tasks.^351^ Solutions to this challenge should focus on better data collection pipelines, as well as making frontier model training more data-efficient.

• Alternative molecular representations. In the domain of MPP, there are alternatives to the textual molecular representations used by LLMs. In particular, graph-based representations such as the molecular graph are used by graph neural networks (GNNs), which are a popular approach to MPP.^352,353^ Indeed, many state-of-the-art GNNs, can achieve comparable or improved prediction accuracy in-comparison to LLMs.^354^ Whilst LLMs have the advantage that they can be applied to domains beyond MPP to assist in areas such as scientific workflows, it remains a challenge for LLMs and frontier models to surpass the predictive performance obtained by models specialised to MPP.

• Advancing multimodal frontier models. Beyond multiple representations of molecules,^305,318–320^ there is a great potential for multimodal frontier models in chemistry to incorporate further related information broadly defined, including lab notebooks, scientific publications, experimental results, images of molecules, spectra information, *etc.*^317^ In fact frontier AI models such as Gemini^355^ and GPT-4^356^ have already incorporated visual, audio and text information to answer complex questions.

• Ethical use & development of frontier models. It is crucial that the development of chemical frontier models follows rigorous scientific process and adheres to research ethical policies. Open science and reproducibility should be promoted *via* suitable open-source practices.^357^ Meanwhile, there must be measures to prevent misuse of frontier models for creating dangerous molecules and materials.^358,359^

## 8 Experimental design for discrete and mixed input spaces

Despite recent successes of large foundation models, many chemistry applications remain challenging due to expensive and difficult data acquisition. In these scenarios, ML-aided experimental design can ensure effective data collection, thereby reducing the number of experiments required.^288^ Bayesian optimisation and active learning are popular approaches for designing experiments.^360–362^ The former aims to optimise the black-box function which is the experiment itself, while the latter seeks to learn the whole function. Both build a surrogate model of the black-box function and then use a decision policy to optimise or learn about the function, respectively. An effective decision policy balances search space exploration and exploitation of the areas expected to be most optimal.^360^

For campaigns to accurately balance exploration and exploitation, the surrogate model needs to incorporate a measure of prediction certainty. This is relatively easy on continuous spaces, as the most popular surrogate models, for example, Gaussian processes, have uncertainty built in ref. 363. However, in many scenarios, input variables are discrete or a mixture of discrete and continuous.^290^ Even within these categories, there is much heterogeneity in the types of discrete variables. This means there is no one-size-fits-all solution to which surrogate model and decision policy should be used, and how uncertainty should be handled. Therefore, selecting an approach reflecting the specific characteristics of the type of variable(s) and the information that can be transferred between variables is essential.

### 8.1 Heterogeneity of problem classes

**Fig. 4:**
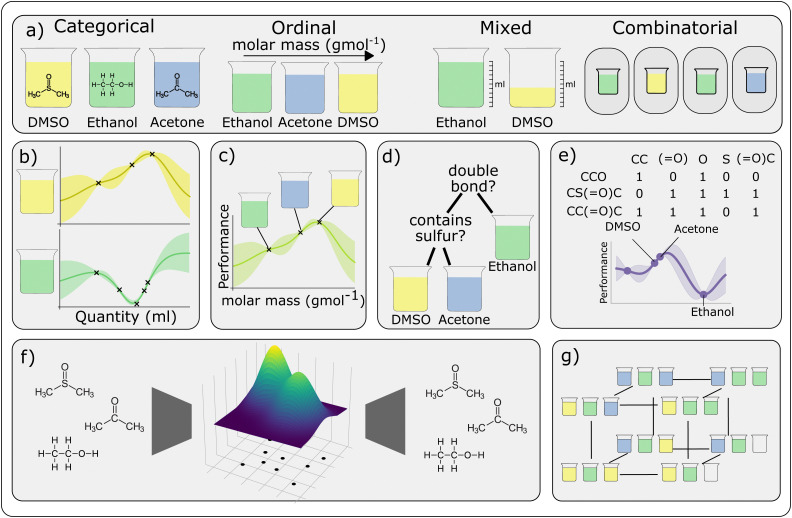
An overview of types of discrete variables (Section 8.2) and surrogate models (Section 8.1). (a) There are four main categories of discrete variables: categorical, ordinal, mixed and combinatorial. (b) Parallel surrogates method, fitting a different surrogate model for each discrete variable. (c) Continuous relaxation where the discrete variables are converted to a continuous one, in this case by using their molar mass. (d) A decision tree-based method where solvents are split into different leaves. (e) String kernel method where the molecules are first converted into SMILES strings, then a string kernel is used to determine their similarity. (f) latent variable methods, where an encoder is used to convert the discrete variable to a continuous latent space, a Gaussian process is fitted to the latent space and optimisation is conducted, then a decoder is used to retrieve the discrete variables again. (g) Graph approach for combinatorial variables, where each node represents a different combination.

One challenge of discrete variables is the diversity of problem types requiring different treatments as shown in panel (a) of Fig. 4. We categorise discrete variables into four types: categorical, ordinal, combinatorial or mixed. Categorical variables take inputs that have no obvious ordering. For example, this might be enzyme cofactors,^364^ solvent type^365,366^ or additives.^367^ A special case of categorical variables are dichotomous variables that can only take two values, such as binary variables,^368^ or on/off. One hot encoding is a technique converting each discrete variable into a unique vector with a single high (1) for the value the variable takes and all other values low (0). This common approach for encoding discrete variables is often used for categorical variables, but can be used for other variable types.^366,369,370^

Ordinal variables are those that can be put in order, such as counts of atoms,^371^ aspect ratios of reactors,^372^ or the number of base-pairs in DNA molecules.^373^ Combinatorial variables take a set of discrete, often finite combinations. Some of the most common combinatorial variables are biological sequences,^374–378^ such as the CDRH3 region of an antibody,^379,380^ or various molecules.^102,381,382^ Combinatorial inputs may or may not have a set length.^379^ González-Duque *et al.*^383^ recently conducted a study comparing many high-dimensional Bayesian optimisation techniques for discrete sequences.

### 8.2 Modelling discrete variables with uncertainty

Another challenge of discrete inputs is determining how much information should be shared between different levels of a discrete variable. For example, if an extra data point is observed that falls in one leaf of a decision tree, how much should that influence the prediction of data points that fall in other leaves? This question has two aspects: how does the new information change the expected value of our prediction and how does it affect the certainty in this prediction? Uncertainty estimates are especially important for experimental design tasks as they guide the exploration of regions where the model is uncertain.

#### 8.2.1 Parallel surrogates

For mixed input problems, a simple way of dealing with discrete variables is to fit a separate continuous surrogate for each value the discrete variables can take, as shown in panel (b) of Fig. 4. When the separate surrogate models are independent, no information is shared. Manson *et al.*^384^ use Gower similarity to measure similarity between data points, essentially based on how many one-hot encodings of their discrete variables are the same.^384^ This approach has been used to optimise chemical reactions by one-hot encoding solvent and ligand variables.^366,385^

It is also possible to learn the similarity between the separate models using transfer learning, such as multi-output Gaussian processes, which learn a covariance function over outputs.^386,387^ This has been used for learning the similarity between cell lines^388^ and DNA molecules. While the parallel surrogates approach is easily implementable, it is generally only feasible for a small number of discrete variables, as the computational cost and amount of data needed scales with the number of values the discrete variables can take.

#### 8.2.2 Continuous relaxation

Another option for ordinal or dichotomous variables is to treat them as continuous but only allow them to take certain variables (panel c) of Fig. 4). This has been used for reactor design,^372^ optimising alloys,^371^ and optimising DNA molecules.^373^ This approach makes handling uncertainty easy as any continuous Bayesian model can be used, most commonly Gaussian processes.^360,363^ However, this can lead to the same points being selected multiple times, especially if all the input variables are discrete and cannot take many values.^389^ This approach is less applicable to categorical or combinatorial variables, as there is no clear ordering of the variables in the continuous space.

#### 8.2.3 Tree models

Tree models offer a natural solution for discrete or mixed inputs as they can create splits on any type of variable,^390^ as shown in panel (d) of Fig. 4. Uncertainty can be incorporated into tree models in a number of ways. Bayesian additive regression trees^391^ offer a way of incorporating uncertainty into trees and have been used for feature selection in catalyst design.^392^ Tree models have also been extended to better model mixed input spaces using Gaussian processes as the leaf nodes.^393^ It has also been shown ensembles of trees are valid kernels for Gaussian processes, counting the similarity between two data points by the number of trees in which they appear in the same leaf.^394–397^

#### 8.2.4 Specialist kernels

A kernel is a function computing the similarity between two data points, enabling algorithms to operate in higher-dimensional spaces without explicitly mapping the data. There is a range of specialist kernels designed for discrete inputs. String kernels^398–400^ measure the distance between two strings based on the number of shared substrings and have been used to optimise molecule design,^382^ see panel (e) of Fig. 4. The transformed overlap kernel measures the Hamming distance between one-hot encodings and has been used for optimising antibodies.^379^ Shortest-path kernels enable global exploration of graph domains.^401^ The Tanimoto kernel^402^ uses Tanimoto coefficients to determine similarity between molecular fingerprints.^367,403,404^ Recently, deep kernels, using the encoder of pre-trained models as a measure between inputs, have been proposed such as the ProteinBERT kernel.^379,405^ Many of these kernels are implemented in GAUCHE, a software package for molecular design.^406^

#### 8.2.5 Latent space

Latent space methods, illustrated in panel (f) of Fig. 4, assume some underlying structure to the discrete variables which means they can be projected onto a lower-dimensional, continuous latent space. This method is particularly popular for combinatorial variables, especially molecule design^102,379,381,407,408^ but can be used for any type of discrete variable. The projection to latent space is usually achieved using a variational autoencoder,^102,367,381,407–409^ but can also be achieved using latent variable Gaussian processes,^373,410–412^ or large pre-trained encoder models such as ChemBERT,^367,413^ UniRep^414^ or ProteinBERT.^405^ Optimisation is then done over the latent space, usually by fitting a Gaussian process to the latent space, which gives a natural estimate of uncertainty. Stanton *et al.*^381^ jointly learn the Gaussian process and encoder to allow for multi-objective Bayesian optimisation of molecules.^381^ A decoder is used to convert the latent variables back to the original form of the discrete variable.

#### 8.2.6 Other approaches

Other methods have been proposed to handle discrete variables. One of these is to build a graph where each node represents a different combination of discrete variable values (panel g) of Fig. 4. This is then optimised using a diffusion kernel.^415,416^ Zhu *et al.*^417^ use a piecewise affine surrogate for a number of chemical experimental design tasks. This has the benefit of allowing for the use of mixed integer programming for optimisation of the target variable and easy handling of constraints.

### 8.3 Decision policies

Parallel surrogates, continuous relaxations and latent space approaches all map discrete variables into a continuous space, enabling the application of well-established continuous experimental design techniques.^366,372,408,409,412^ These methods typically employ a surrogate model, normally a Gaussian process, to estimate the mean response and the associated uncertainty. An acquisition function then combines the mean and uncertainty into a single metric guiding experimental design. For Bayesian optimisation, where the aim is optimising a target variable, common acquisition functions are expected improvement and upper confidence bound.^360,361^ Other experimental design strategies include active learning, which aims to learn the whole function,^362^ and Bayesian quadrature, which seeks to learn an integral.^418^

Applying continuous optimisation methods to ordinal variables and selecting the closest integer value can lead to the same points being repeatedly sampled, wasting the experimental budget. This can be compensated for by altering the acquisition function when this occurs,^389^ or transforming the inputs before calculating the acquisition function.^419^ Continuous methods can be applied to parallel surrogate models, although this gets expensive when there are many continuous spaces or the continuous spaces are high dimensional. To reduce this computational cost, multi-armed bandits can be used to select which surrogates are most likely to offer improvements.^420,421^ Latent space approaches generally assume smoothness,^381^ allowing for Bayesian optimisation or active learning. In these cases, a Gaussian process is fitted to the latent space, where the output of the Gaussian process is the objective function.^407,408^ If the latent space is high dimensional, trust-region methods can be used to guide exploration of the space.^408^

For methods that do not convert discrete variables to continuous inputs, the biggest challenge is often exploring the space, as gradient-based optimisation methods can no longer be applied to the acquisition function. In combinatorial spaces, evolutionary or random walk algorithms can be used. For example, Khan *et al.*^379^ use random walk to explore a trust region, evaluating the acquisition function at each point.^379,422^ Bayesian optimisation for tree models can be done by optimising each leaf of the tree and picking the best one,^423^ using local search where a step is taken in one parameter at a time^424^ and global optimisation of the acquisition function using mixed integer programming.^425^

When specialist kernels, such as string kernels and Tanimoto kernels are used, genetic algorithms can explore the search space.^382^ Recent work has also demonstrated how transformer neural processes, a *meta*-learning model, where models use knowledge from previous datasets to learn a new task, can skip fitting a surrogate and directly *meta*-learn the acquisition model.^380^

### 8.4 Outlook

Experimental design over discrete and mixed inputs is challenging due to the heterogeneity of problem types, difficulty modelling uncertainty and lack of gradients for optimising acquisition functions. To mitigate these challenges, it is important to identify the types of discrete variable(s) present in a problem and select the right surrogate model. The methods outlined here have all been proven to work for several chemistry applications, however, uptake of such methods is slow. Several software packages have been developed to help experimentalists apply these approaches to their experimentalists: BOtorch,^426^ BOFire,^427^ and BayBe^428^ are all Bayesian optimisation packages; WebBO^429^ is a modular platform that can be integrated into electronic lab book frameworks; Atlas,^430^ Anubis,^431^ and Dragonfly^432^ are all packages for self-driving labs that integrate experimental design methodologies. To ensure its proper use it is important software incorporates educational aspects that help experimentalists, who may not be well versed in ML, to map their problem to the available methods and understand the assumptions being made.

From a methodological perspective, future research directions include *meta*-learning of the acquisition function to amortise inference and to skip the need for a surrogate model altogether,^380,433^ dealing with systems where decisions that change with time^434^ and using experimental design to uncover causal relationships.^435–437^

## 9 AI for robotics in chemistry

Traditional chemistry laboratories rely heavily on human labour for repetitive, time-intensive, and sometimes hazardous tasks, such as chemical synthesis, sample preparation, and data analysis.^438^ This reliance on manual processes not only reduces operational efficiency but also exposes scientists to potentially harmful environments. The integration of robotics and automation into laboratory environments has emerged as a promising solution, which enables improved process optimisation, greater precision, and the potential for continuous operations without human intervention.

Recent advancements in AI, combined with access to large-scale datasets and sophisticated laboratory automation tools, such as systems for synthesis, separation, purification, and characterisation,^439^ have enabled the development of ‘robot chemists’. These systems utilise AI as the cognitive engine, empowering robotic platforms to autonomously conduct experiments and transform traditional workflows in chemistry. AI-driven robotics are transforming laboratory practices by addressing inefficiencies and introducing advanced capabilities that streamline scientific research. These systems optimise workflows through continuous, autonomous operations, significantly reducing the time required for experimental iterations while enhancing productivity far beyond human limitations. By standardizing processes and minimising errors, they ensure consistent, reproducible results, fostering greater confidence in experimental outcomes. A key advantage of AI-driven robotics is their ability to handle hazardous chemicals and conduct high-risk reactions, thereby safeguarding human researchers and mitigating safety risks. Moreover, these systems excel in scalability, making them invaluable for large-scale research endeavours such as high-throughput screening and combinatorial studies. They could theoretically manage vast experimental conditions with remarkable speed and accuracy, enabling the exploration of expansive chemical and parameter spaces that would be infeasible manually.

These advancements, when adopted and employed, have effectively transformed traditional laboratories into automated discovery platforms, thereby significantly increasing the autonomy of scientific experimentation. The integration of ‘robot chemists’ (systems capable of automated learning, reasoning, and experimentation), has accelerated the discovery of new molecules, materials, and systems. By leveraging diverse data sources and modalities, these intelligent systems are able to operate continuously, make decisions under uncertainty, and generate reproducible data enhanced with comprehensive metadata and real-time sharing capabilities. This paradigm shift not only improves precision, efficiency, and scalability but also minimises manual errors and broadens the generalisability of research across a wide range of applications.^438^ Here, we discuss the prospective impact of integrating AI and robotics in chemistry. We begin by classifying ‘Robotic Chemists’ based on their levels of autonomy and highlighting their contributions to self-driving laboratories (SDLs). Finally, we outline a future roadmap for the development of AI and robotics in chemistry.

### 9.1 Classification of ‘robotic chemists’ based on autonomy levels

**Fig. 5:**
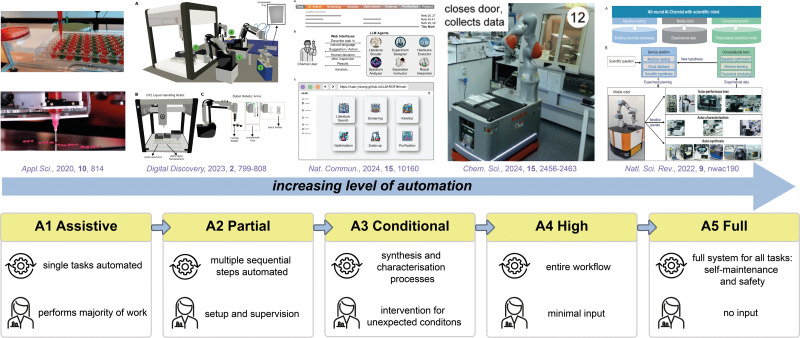
The five levels of autonomy differ in the automated steps and the level of human intervention. Select examples are depicted spanning the range of automation spanned by the levels of autonomy. Figures in the top panel are reproduced under a Creative Commons license.^445,447–450^

There are five levels of autonomy: (i) assistive automation, (ii) partial automation, (iii) conditional automation, (iv) high automation, and (v) full automation. Here, we discuss each level of autonomy as it relates to self-driving laboratories. Table 1 and Fig. 5 present an overview of the levels of autonomy and concrete examples in chemistry.

**Table 1:** Classification of intelligent robots in chemistry by autonomy level

Autonomy level	Description	Example	Ref.
A1: Assistive automation	Automates single tasks; humans perform the majority of work.	Automated liquid handling systems that perform repetitive aspiration and dispensing tasks, reducing manual labour and minimising errors.	440
A2: Partial automation	Automates multiple sequential steps; requires human setup and supervision.	Setups where robotic arms handle the transfer of reactants between different stages of a reaction sequence, creating a distributed automation system.	441
A3: Conditional automation	Fully automates synthesis and characterisation processes; human intervention needed for unexpected conditions.	The ‘RoboChem’ platform developed by the University of Amsterdam autonomously performs chemical syntheses and optimises reaction conditions using AI-driven ML.	442–444
A4: High automation	Automates entire workflows, including setup and adaptation to unusual conditions; minimal human input.	The mobile robotic chemist developed by the University of Liverpool autonomously navigates laboratory environments and conducts experiments across various areas of chemical synthesis.	343,445,446
A5: Full automation	Completely autonomous systems capable of handling all tasks, including self-maintenance and safety hazard resolution.	This is an active area of research within chemistry, and will be powerful for chemical tasks where human input is not necessary.	

#### 9.1.1 A1: Assistive automation

This represents the initial stage of laboratory automation, where individual tasks are automated to support human operators who still perform the majority of experimental workflows. The focus at this level is on alleviating repetitive and time-consuming tasks, thereby improving efficiency, precision, and consistency while freeing researchers to focus on more complex aspects of experimentation. A key example of assistive automation is automated liquid handling systems which perform tasks such as aspirating, dispensing, and plate replication with high precision.^440^ These systems are indispensable in applications like high-throughput screening and sample preparation, managing thousands of samples with minimal human intervention and significantly reducing the potential for human error.

#### 9.1.2 A2: Partial automation

This involves the integration of multiple sequential tasks within a single laboratory workflow. At this level, systems can perform a series of operations autonomously, but require human input for setup, oversight, and occasional intervention. Partial automation aims to streamline workflows, reduce manual effort, and enhance overall efficiency without fully eliminating the need for human involvement. An example of this would be a dual-arm robot being used to transfer samples between stations for purification and analysis, as well as to open and close individual devices within a pipeline for bioanalytical applications.^451^ This integration is particularly valuable in pharmaceutical research, where it accelerates structural analysis and ensures consistency in sample handling.^441^

#### 9.1.3 A3: Conditional automation

This represents a stage where synthesis and characterisation processes are fully automated, requiring human intervention only in response to unexpected conditions. At this level, robotic systems autonomously execute predefined tasks, leveraging AI and advanced sensors to adapt to routine variations but relying on human oversight to resolve anomalies or unforeseen challenges.

The RoboChem platform,^442^ developed by the University of Amsterdam, is an autonomous chemical synthesis robot that incorporates an AI-driven ML module. This platform can autonomously conduct chemical syntheses, optimise reaction conditions, and iteratively refine processes with minimal human involvement. The RoboChem platform has demonstrated superior performance compared to human chemists in terms of speed and accuracy, significantly accelerating the discovery of new molecules for pharmaceutical and industrial applications. The AlphaFlow system,^443^ designed to handle multistep synthesis and characterisation in flow chemistry, utilises reinforcement learning to optimise reaction pathways, monitors real-time data, and adjusts parameters to enhance efficiency and reproducibility. By integrating ML with advanced automation, AlphaFlow exemplifies the potential of conditional automation to streamline complex chemical processes. Another example is the ORGANA robotic assistant, a system designed to automate a wide range of chemistry experiments. ORGANA leverages LLMs to plan and execute experiments, interact with chemists using natural language, and adapt to diverse experimental requirements.^444^

#### 9.1.4 A4: High automation

This represents a significant advancement in robotic autonomy, where systems are capable of automating entire workflows with minimal human input. This includes experimental setup, execution, and adaptation to unusual conditions or unexpected challenges. At this level, robots can handle complex tasks autonomously, requiring human intervention only for high-level oversight or strategic decision-making. A notable example of high automation is the mobile robotic chemist developed by the University of Liverpool.^446^ This system is designed to autonomously navigate laboratory environments, identify experimental opportunities, and conduct a diverse range of experiments across various domains of chemical synthesis. Its mobility and ability to integrate AI-driven decision-making enable it to adapt to different laboratory layouts and experimental requirements, making it a versatile tool for advancing research. The Coscientist represents another example, which can autonomously conduct complex tasks like synthesizing molecules, optimising reactions, and programming robotic systems to perform specific experimental protocols.^343^ Equipped with a computational brain, machine reading capabilities, and mobile robotic modules, an AI-Chemist seamlessly integrates literature-based knowledge extraction,^445^ automated synthesis, characterisation, and performance testing. Through closed-loop operations powered by ML and Bayesian optimisation, it can achieve a level of precision and adaptability that surpasses human capabilities.

#### 9.1.5 A5: Full automation

This represents the highest level of robotic autonomy in chemistry laboratories, characterised by complete independence in managing all experimental tasks, including safety hazard resolution and self-maintenance. At this stage, systems are capable of synthesising, characterising, and iteratively optimising chemical processes without human involvement. Fully autonomous cloud laboratories exemplify this level of automation, integrating artificial intelligence and robotic systems to design, execute, and analyse experiments remotely. Self-maintenance capabilities, such as routine calibration, cleaning, and predictive maintenance, further enhance these systems’ operational reliability.

### 9.2 Applications of self-driving laboratories

AI-driven robotics are making significant contributions to labs in chemistry, materials science, and biochemistry, such as complex reaction optimisation, high-throughput screening, and hazardous material handling. More specifically, advancements in AI and robotics are reshaping laboratories into self-driving labs (SDLs),^452^ which support advanced scientific discovery with minimal human intervention. Globally, SDLs are under active development in numerous laboratories, certainly too many to name here. SDLs are also described as materials acceleration platforms,^453^ Lab 4.0,^454^ the Internet of Laboratory Things,^455^ Robot Scientists,^456^ the autonomous research system (ARES),^457^ and autonomous experimentation systems.^458^ SDLs can conduct experimental design and execution, real-time data analysis, and parameter optimisation in an iterative process. This approach consists of three core components: a robotic system to conduct and analyse reactions, software to interpret analytical data, and an algorithm that correlates reaction outcomes with experimental parameters. The essence of SDLs lies in their ability to run closed-loop experiments, which utilises online analytics, real-time feedback from each experiment, and decision-making algorithms to inform subsequent actions.^459–461^

Unlike conventional, human-dependent laboratories in chemistry and materials science, SDLs overcome three major limitations of traditional laboratories: (1) the slow and inefficient exploration of experimental space; (2) the lack of integration across different experimental stages; and (3) delays between the completion of one experiment and the initiation of the next.^462^ Here, some typical examples of applications are summarised, including high-throughput experimentation (HTE), self-optimising reaction systems, automated discovery platforms, and protein engineering. Importantly, there are many additional examples across chemistry.

HTE aims to rapidly screen and analyse chemical compounds through advanced automation and AI integration. One example is the HTE OS framework, developed specifically for robotic platforms, demonstrates the potential of AI-driven experimentation.^463^ By combining advanced scheduling algorithms, data processing techniques, and natural language processing (NLP) tools, these systems achieve parallel experimentation, significantly reducing the time required to evaluate large chemical libraries or complex reaction matrices.^464^ Autonomous robotic platforms can also be tailored for specific tasks, for example, for electrolyte formulation and coin cell assembly in high-throughput lithium-ion battery research.^448^ Additionally, high-throughput synthesis (HTS) enhances throughput by enabling researchers to synthesise multiple materials simultaneously through automated parallel processing.^465^

Self-optimising reaction systems leverage AI algorithms to dynamically adjust reaction parameters in real time, optimising critical outcomes such as yield or selectivity. Both autonomous and semi-autonomous robotic systems have contributed to the development of novel chemical synthesis methodologies.^344,443,460,462^ One example is from Schwalbe-Koda *et al.*,^466^ who describe a platform that autonomously optimises polymer synthesis using self-optimising flow reactors. These reactors iteratively adjust key variables, such as temperature and pH, based on real-time reaction monitoring. This approach has been further refined for applications such as photoinitiated RAFT polymerisation, where fully automated systems leverage real-time feedback to enhance process outcomes continuously.^467^

Data-driven ML algorithms are transforming materials and catalyst discovery, enabling the rapid analysis of experimental data to identify optimal candidates or refine reaction conditions. These systems autonomously explore novel chemical transformations, accelerating reaction discovery while minimising risks associated with reactive materials. Robotic systems, operating under optimised safety protocols, enable safe and efficient experimentation. For instance, the ‘Schlenkputer’ system executes reactive chemical transformations autonomously, employing AI algorithms to prioritise experimental pathways based on predicted reactivity.^468^

Protein engineering holds significant potential for applications in chemistry. However, the development of new proteins with enhanced or novel functions has traditionally been slow, labour-intensive, and inefficient. The Self-driving autonomous machines for protein landscape exploration (SAMPLE) platform represents a breakthrough in this domain. This fully autonomous system integrates an intelligent agent that analyses protein sequence-function relationships, generates new protein designs, and coordinates with an automated robotic system to experimentally test these designs. Feedback from the robotic system enables the intelligent agent to refine its understanding and optimise the protein engineering process iteratively.^378^

### 9.3 Future research directions

#### 9.3.1 Open-source tools and hardware

Open-source tools and hardware play an essential role in democratising access to automated chemistry by lowering entry barriers. For instance, Opentrons offer low-cost open-source liquid handling platforms with a Python API which have been utilised more and more in chemistry,^448,449,469,470^ and the development of Chemspyd, an open-source Python interface for Chemspeed robotic platforms,^471^ allows real-time adaptive control over automated platforms and integrates with the scientific Python ecosystem. Chemspyd also includes a natural language interface that generates Chemspyd code through iterative GPT-4 prompting. Open hardware tools like the digital pipette^472^ also offer customisable solutions for liquid transfer, empowering researchers to create and adapt their own automated systems.

#### 9.3.2 Cloud laboratories

Cloud laboratories are transforming scientific research by enabling remote, AI-powered experimentation. These platforms allow scientists to design, execute, and analyse complex experiments without being physically present in traditional laboratory settings, thereby democratising access to advanced laboratory automation and fostering global collaboration. A notable example is the Emerald Cloud Lab (ECL),^473^ which operates a fully automated life sciences laboratory. Researchers can remotely conduct wet-lab experiments by sending samples to ECL's facility and designing experiments through a command-based software interface. This setup facilitates continuous operation of multiple complex workflows, enhancing efficiency and productivity.

#### 9.3.3 Development of modular, scalable, cost-effective and accessible systems

Most automated laboratory setups depend on specialised equipment and complex integrations of equipment from multiple suppliers, often requiring advanced technical skills and programming capabilities, which can restrict accessibility. To encourage broader adoption, it is essential to develop standardised architectures that seamlessly integrate robotic and laboratory equipment in a user-friendly manner that can adapt to diverse experimental needs.^474^ Modular SDLs enable different robots to perform specific tasks within a workflow, allowing for scalability and flexibility as robotic units can be added or modified according to evolving laboratory requirements. However, despite significant advancements in SDL technology, challenges remain in creating standardised, cost-effective hardware and accessible software solutions. Inspired by the concept of the digital twin—a virtual representation of a physical object—researchers have introduced the ‘frugal twin’, a low-cost alternative to high-end SDLs.^475^ These low-cost SDLs, or frugal twins, costing under 1000 USD, offer a balanced trade-off between cost and functionality, making them ideal for educational and research environments where affordability is essential.^459^

#### 9.3.4 Integration of advanced simulation tools

The adoption of advanced 3D simulation tools is essential for modelling complex processes such as liquid handling, thermal fluctuations, and chemical reactions prior to real-world experimentation. These tools enhance safety, enable rapid workflow prototyping, and reduce risks, particularly when dealing with hazardous materials. For instance, Chemistry3D,^476^ developed on NVIDIAs Omniverse platform, allows researchers to simulate robotic operations and chemical processes within an interactive 3D virtual environment. It delivers real-time feedback on key parameters such as temperature, colour, and pH, enabling more informed and precise decision-making.

#### 9.3.5 General-purpose robots empowered by LLMs

Natural language interfaces represent a significant advancement in simplifying robotics for non-experts by enabling intuitive interaction with complex systems. For instance, CLAIRify effectively combines iterative prompting with program verification to translate natural language commands into executable robotic instructions. This approach addresses the challenging task of converting user instructions into robotic actions while ensuring adherence to safety constraints.^477^ By leveraging solvers like PDDLStream, CLAIRify generates workflows that are both safe and syntactically correct, which can mitigate risks such as spills and collisions. Similarly, advancements in robotic chemistry, such as the development of a universal chemical programming language, aim to enhance the repeatability and standardisation of robotic synthesis. Proposed by Benini *et al.*, this language facilitates seamless protocol execution across diverse robotic platforms.^463^ A notable innovation is *χDL*, a platform-agnostic and machine-readable chemical description language. By encoding and enabling the execution of synthesis protocols across different systems, *χDL* significantly enhances laboratory automation and interoperability.^463^ These innovations are poised to bridge the gap between human intent and robotic execution in diverse scientific and industrial applications.

As discussed in the earlier sections, LLMs are further transforming the field of chemistry by extracting and interpreting complex chemical information from vast scientific literature. The integration of advanced multi-language large models (MLLMs) into robotics offers significant potential for laboratory automation. These models enhance the adaptability of robotic systems, allowing them to address diverse research challenges. To make these systems accessible to chemists, who often lack robotics expertise, there is a focus on creating user-friendly tools that simplify programming, data analysis, and experimental setup adaptation.^474^

#### 9.3.6 Collaborative human-AI systems

The future of laboratory research lies in the seamless collaboration between human scientists and AI-driven robotic systems. In this model, researchers can focus on addressing complex, high-level scientific challenges while delegating routine or intricate tasks to robotic systems.^478^ This human-in-the-loop approach ensures that scientists maintain oversight and control of experimental processes, leveraging the precision, scalability, and efficiency of automation without compromising on adaptability or creativity.^479^

Mixed-use laboratories, where humans and robots work side by side, will set new standards for safety and efficiency. These environments will integrate advanced monitoring systems and adaptive technologies to ensure secure and harmonious operations. Intuitive interfaces—such as voice commands and generative AI tools—will further enhance the accessibility and usability of automated systems, enabling smoother interactions and fostering a productive partnership between humans and machines.

## 10 AI-accelerated data management for digital chemistry

The proliferation of AI- and data-driven approaches in the physical sciences have the potential to not only accelerate scientific discovery, but also allow us to tackle qualitatively different problems. However, in order to really exploit this potential, we need to significantly improve the quality, quantity, and accessibility of the data captured in the modern research lab. In a recent survey by the UK's Physical Sciences Data Infrastructure (PSDI), less than 20% of respondents digitally managed all of their laboratory data and experiments.^480^ Among those, a variety of software packages were used, with varying levels of machine accessibility to the data. Ultimately, the large majority of laboratory data currently produced is not stored in such a way that it can be readily actioned upon by AI tools. There is, therefore, a timely need for data infrastructure that can help researchers to capture, organise, and share their data along with its provenance, metadata, and scientific context. Here, we discuss current and projected capabilities of laboratory data management for AI, and discuss our own efforts to integrate both AI assistants and agents into experimental materials chemistry research within the open source^481^*datalab* electronic laboratory notebook platform. We envision that the development and adoption of interoperable data management platforms that reproducibly store and make available diverse laboratory data will be necessary to reach the full potential of AI tools for scientific research.

### 10.1 The role of AI-powered assistants and agents

As the quantity and diversity of our scientific data grows, researchers find themselves spending an increasing amount of time and effort managing data: organising connected experiments, converting between file formats, and performing analysis.^480,482,483^ The recent advent of capable ML models, in particular multi-modal large language models^291,292,484–486^ provides an extraordinary opportunity – for researchers and tool-builders alike – to build capable AI-driven agents and assistants that can meaningfully accelerate science by aiding experimental researchers in these data management and analysis tasks.^487,488^ Today's LLM-based tools generally fall into two categories: assistants and agents. Assistants, typified by the first iteration of ChatGPT released in November 2022, present the user with a chat-based interface to an (M)LLM that can answer user questions and perform basic tasks. The data for an assistant can either be pre-loaded directly into the prompt, or can be fetched as needed using search tools (*i.e.*, Retrieval-augmented generation, RAG). On the other hand, autonomous LLM agents go a step further by allowing the LLM to iteratively take actions, observe the outcome, and then react further to accomplish a task. For example, an AI agent may have the ability to access web APIs, write arbitrary code, execute it, parse the output, and perform further actions. In principle, an AI agent could complete very complex research data management and analysis tasks that require multiple conversions, comparisons, visualisation, and information synthesis. However, while LLM-based assistants are currently well established, truly capable general-purpose agents are still early in their development.

Both assistants and agents have the potential to greatly aid in scientific laboratory research. Assistants may read large quantities of (potentially multimodal) laboratory data from machine-accessible electronic laboratory notebooks in order to quickly summarise results, draw connections, extract data, or even propose new experiments. Assistants can also integrate various third-party tools to perform queries and simple analysis.^489^ Agents, on the other hand, can be tasked with more complex data-handling tasks that require multiple steps. For example, a researcher developing new battery electrodes may pose the following prompt: “Based on all the cathodes developed in this lab in the last 5 years, determine whether there is a correlation between particle size (by scanning electron microscopy (SEM) analysis) and battery performance”. While this question would be very time-consuming for a human researcher answer, a capable AI agent may attempt to tackle this problem autonomously by (1) writing code to search the groups electronic notebook for all relevant samples, (2) using vision capabilities, or specialist software provided in a machine-actionable way to the agent,^490^ to view SEM images from these samples, (3) writing code to analyse for any correlations and create a useful visualisation for the researchers. In this way, an AI agent could dramatically speed up human-driven research, by allowing researchers to quickly and easily ask important scientific questions that were inaccessible before.

### 10.2 The importance of user interfaces for data capture

The full utility of AI assistants and agents will only be realised if the scientific data, metadata, protocols, and observations that we collect daily in the lab are stored in a manner where they can easily be accessed by machine agents. For example, our open-source laboratory data management platform, *datalab*,^481^ stores scientific data along with relevant metadata and context in a database, and provides both GUI (human-friendly) and API (machine–friendly) interfaces. Within the GUI, a LLM-powered assistant, “whinchat”,^487^ can read, summarise, and answer questions about the recorded experiments. For more complex data management tasks, we have developed an external AI-powered agent, “yeLLowhaMmer”.^488^ YeLLowhaMmmer is pre-prompted with the *datalab* API documentation, so that it can iteratively write and execute Python code to access, filter, and process data as needed. A future area of development is capturing and storing the results of AI queries and automated analysis so that it can be reused and shared across a lab. It will be especially important to mark AI-generated content as such, so that it can be appropriately verified by humans.

As we have discussed, the usefulness of AI-based tools hinges on the availability of scientific data. To make use of AI-based tools, laboratory data should be stored digitally, with all the metadata and context needed to make it experimentally useful. Researchers should strongly consider open, machine-accessible formats^483^ – ideally on a platform that allows programmatic access. No single platform or data management strategy will suit all research use cases, but developers should prioritise those with open APIs, schemas and code to enhance interoperability among tools.^482,491–496^ In our own work, we have found that a “semi-normalised” data model provides the best balance between rigidity and flexibility for laboratory work and for interaction with LLMs. Data sets are recorded with schemas that specify common fields and their data types, but free-text fields are also provided so that users can easily record information or observations that do not fit neatly into the predetermined schemas. From this base, LLMs will likely also find use in mapping to richer semantic data formats that can be used to readily exchange data in an interoperable way for use in knowledge graphs and other applications.^496,497^ Importantly, the use of machine-accessible data management platform not only enables the use of state-of-the art AI tools in experiments, but also makes it possible for researchers to contribute their data to train or fine-tune the next generation of ML models, if they should choose to share their data in this manner.

## Future outlook

Retrospective benchmarks that reliably predict prospective success are necessary to improve the efficiency of AI-driven discovery, including drug discovery. Indeed, these advancements will also have implications for the application of generative models for chemical discovery. While few studies have experimentally validated high-performing compounds proposed by generative models, these methods have already demonstrated their unique ability to inspire human creativity. Similarly, retrosynthesis tools face challenges related to reliability, route selection, data quality, and adoption – motivating additional research in these areas.

Beyond retrosynthetic planning, frontier models, including LLMs, are set to play an important role in experimental workflows. Yet, there exist several challenges to overcome, including mitigating hallucinations, advancing data-efficient raining, advancing multimodal models, and ensuring ethical use of frontier models in chemistry. Indeed, integrating LLMs in chemical workflows extends to their use in robotics and automation equipment.

The role of automation in experimental chemistry is continuing to improve. We note that, while there is a general move towards fully automated setups, that the value of human input and intervention should not be underestimated. Indeed, human-in-the-loop initiatives leverage the productivity of robotic automation, the efficiency of autonomous decision making, with the insight of human chemists. Future progress will depend on the development of: (i) open source tools; (ii) modular and scalable systems; (iii) cost-effective and accessible platforms; and (iv) advanced human-AI collaborative systems. Beyond these advancements, additional development of sensors and chemometrics that facilitate *in situ* analysis that does not require additional units of operation are paramount. Indeed, while most procedures can already be highly automated, it is the analytical tools that provide the data needed for ML. High-throughput analysis poses a challenge – while it can be fairly easy to automate sample preparation, a large proportion of characterisation techniques are still carried out offline, albeit equipped with autosamplers for handling larger numbers, and still carry out measurements in a sequential one-by-one manner which can be time-consuming. In addition to robotics and frontier models, AI-driven decision making algorithms have also started to redefine how experiments are planned and executed. Improvements in each of these areas are paramount to realising a fully autonomous chemical research workflow.

Underpinning all of these advancements is the critical need for accessible and robust data infrastructure, facilitating the creation of high-quality scientific data. The success of any AI model is dependent on the quality of the data on which it is trained. Thus, efforts to unify metadata standards, improve open-access data repositories and databases, as well as initiatives to ensure that laboratory data are stored digitally with associated metadata and made available in machine-readable formats through open platforms and APIs is critical to advancing AI-driven chemical research.

There is, undoubtedly, immense potential of AI to accelerate chemical research. However, realising the full impact requires addressing technical, methodological, and physical challenges. Sustained interdisciplinary collaboration and a commitment to open science and discourse are necessary to overcome these challenges, and advance both fundamental understanding of chemical phenomena and acceleration of fundamental research to real-world applications.

## Author contributions

J. M. F. contributed AI for Quantum Chemistry. I. M. L. and A. W. contributed Scaling Atomistic Simulations with ML Force Fields. A. M. G. contributed Generative AI. P. J. B. contributed AI for Drug Discovery. F. H. and E. A. D. R. C. contributed Synthesis Route Planning and Selection *via* Deep Learning. K. K. H. contributed Data-rich and Data-led Experimentation to Support Development and Accurate Predictive Models. I. S. J. and Y. L. contributed LLMs and Multimodal Models for Chemistry. R. S. and R. M. contributed Experimental Design for Discrete and Mixed Input Spaces. D. Z. wrote the first draft of AI for Robotics in Chemistry. A. R. B. and R. L. G. finalized the AI for Robotics in Chemistry section. M. L. E. and J. D. B. contributed AI-Accelerated Data Management for Digital Chemistry. A. M. M. co-conceived the article focus and collated and organized the sections within the manuscript, and contributed the first draft of the introduction and conclusions. K. E. J. co-conceived the article and organized topics. All authors contributed to the editing of the final version of the manuscript.

## Data availability

No primary research results, software or code have been included and no new data were generated or analysed as part of this review.

## Conflicts of interest

M. L. E. is a shareholder and Director of datalab industries ltd.
